# Regional variation of COVID-19 admissions, acute kidney injury and mortality in England - a national observational study using administrative data

**DOI:** 10.1186/s12879-024-09210-6

**Published:** 2024-03-22

**Authors:** Nitin V Kolhe, Richard J Fluck, Maarten W Taal

**Affiliations:** 1 University Hospitals of Derby and Burton NHS Trust, Uttoxeter Road, Derby, DE22 3NE UK; 2 Centre for Kidney Research and Innovation, Academic Unit for Translational Medical Sciences, School of Medicine, University of Nottingham, Nottingham, UK

**Keywords:** COVID-19, Epidemiology, Variation, Acute kidney injury

## Abstract

**Background:**

This study explores regional variations in COVID-19 hospitalization rates, in-hospital mortality, and acute kidney injury (AKI) in England. We investigated the influence of population demographic characteristics, viral strain changes, and therapeutic advances on clinical outcomes.

**Methods:**

Using hospital episode statistics, we conducted a retrospective cohort study with 749,844 admissions in 337,029 adult patients with laboratory-confirmed COVID-19 infection (March 1, 2020, to March 31, 2021). Multivariable logistic regression identified factors predicting AKI and mortality in COVID-19 hospitalized patients.

**Results:**

London had the highest number of COVID-19 admissions (131,338, 18%), followed by the North-west region (122,683, 16%). The North-west had the highest population incidence of COVID-19 hospital admissions (21,167 per million population, pmp), while the South-west had the lowest (9,292 admissions pmp). Patients in London were relatively younger (67.0 ± 17.7 years) than those in the East of England (72.2 ± 16.8 years). The shortest length of stay was in the North-east (12.2 ± 14.9 days), while the longest was in the North-west (15.2 ± 17.9 days). All eight regions had higher odds of death compared to London, ranging from OR 1.04 (95% CI 1.00, 1.07) in the South-west to OR 1.24 (95% CI 1.21, 1.28) in the North-west. Older age, Asian ethnicity, emergency admission, transfers from other hospitals, AKI presence, ITU admission, social deprivation, and comorbidity were associated with higher odds of death. AKI incidence was 30.3%, and all regions had lower odds of developing AKI compared to London. Increasing age, mixed and black ethnicity, emergency admission, transfers from other providers, ITU care, and different levels of comorbidity were associated with higher odds of developing AKI.

**Conclusions:**

London exhibited higher hospital admission numbers and AKI incidence, but lower odds of death compared to other regions in England.

**Trial registration:**

Registered on National Library of Medicine website (www.clinicaltrials.gov) with registration number NCT04579562 on 8/10/2020.

**Supplementary Information:**

The online version contains supplementary material available at 10.1186/s12879-024-09210-6.

The study is registered on the National Library of Medicine website (www.clinicaltrials.gov) on 08/10/2020 with registration number NCT04579562.

## Introduction

The COVID-19 pandemic overwhelmed global healthcare systems and exposed social and geographic inequities. Regional variations were observed, attributed to population density and characteristics, impacting infection and hospitalization rates [1–3]. Racial disparities and social inequalities were evident in infections, hospitalizations, and mortality rates. Prevention strategies, such as lockdowns, mask-wearing, and vaccination, played a role in limiting the virus spread and hospitalizations [2, 4–6]. In England, in the early part of the pandemic, mortality in the most deprived areas was double that observed in the least deprived areas [6]. In addition, early studies revealed racial inequality with a disproportionate increased mortality rate in Black and Asian populations [7–9]. More recent studies have brought to light that these disparities encompass significant variations in exposure risk and, after hospitalization, some differences in prognosis [10]. This was not confined to England alone but was also reported in studies from the United States, where positive correlations existed between COVID-19 deaths and the proportion of Black or Asian residents in counties [11]. The dynamics of transmission has been linked, not only to virulence of the SARS CoV-2 strains, but also to prevention strategy. Prevention strategies in the form of lock downs, mask-wearing and vaccination may be useful to limit the spread of virus and resulting hospitalization. However, the prevention strategies may vary from region to region within a country and this may affect hospitalizations and outcomes.

In England, national lockdown measures came into force legally on 26th March 2020, and were downgraded from 4th July 2020. There was subsequent introduction of local restrictions in the Midlands and North of England. A further tiered approach to restrictions was applied in October 2020 in response to increasing COVID infections and resultant hospitalizations in Northern England. The local restrictions, introduced by the government, were aimed at disease containment, and minimising the severe economic effect of national lockdowns [12].

In June 2020, the publication of the RECOVERY trial provided evidence for benefit from steroid treatment and led to a change in practice which may have resulted in reduced adverse outcomes from SARS CoV-2 infection. However, clinical practice may have varied depending on the regional capacity to learn and react. In view of differences in population density, deprivation, regional lockdowns, and prevention strategies, it is important to understand regional variation in hospitalization and outcomes of COVID-19 in England. Though short-term studies in the early part of the pandemic had shown regional variation in SARS CoV-2 infection and mortality related to deprivation and ethnicity, only a handful studies have used national hospital admissions data to study regional variation of COVID-19 hospitalization and mortality over a longer period [4, 6, 13–15]. Furthermore, previous studies on outcomes associated with COVID hospital admissions have tended not to include the impact of acute kidney injury (AKI), a complication reported to be associated with a marked increase in risk of death [4, 16, 17].

This study represents the first comprehensive regional analysis of confirmed COVID-19 hospitalization data and impact of AKI for the whole of England, a country severely impacted by COVID-19, and benefits from comprehensive data collection through a unified healthcare system, the National Health Service. The aim of this study was to describe regional variation in COVID-19 hospitalization and mortality and evaluate the determinants of in-hospital mortality including AKI. We hypothesized that London, with its higher population density and socio-cultural diversity, would have higher in-hospital mortality in patients with COVID-19.

## Methods

### Study design and ethical approval

This was an investigator-initiated, retrospective cohort study using the hospital episode statistics (HES) - English National database. The study protocol was assessed by the Research and Development Department of University Hospitals of Derby and Burton (UHDB) National Health Service (NHS) Trust and approved by the Health Research Authority and Wales Research Ethics Committee. It was registered on the National Library of Medicine website (www.clinicaltrials.gov) on 08/10/2020 with registration number NCT04579562. The protocol is available in supplementary file (S1 Text). Data were analysed and interpreted by the authors who reviewed the manuscript and confirm the accuracy and completeness of the data and adherence to the protocol. The study was conducted according to the principles expressed in the Declaration of Helsinki, and the results are reported according to the strengthening the reporting of observational studies in epidemiology (STROBE) guidelines.

### Study design and procedures

We used the Hospital Episode Statistics (HES) data warehouse to extract the data of all adult patients, 18 years of age and upwards [those with a birth date on or before 01/03/2002], who were admitted to the hospital with COVID-19 infection between 1st March 2020 and 31st March 2021, to the end of the discharge period and included the diagnostic code for COVID-19 (U07.1) in any of the 20 diagnosis codes. The HES database collects detailed records of all patients admitted to any hospital in England and is commissioned by National Health Service (NHS). The data in HES are recorded at each finished consultant episode (FCE) level, which represents the care delivered by a single consultant. A “spell” represents a complete admission, and each finished spell or admission may contain more than one FCE. FCEs and spells are susceptible to variations in the way hospitals organise their care, and in particular their propensity to transfer patients between consultants or to other hospitals. We linked the dataset from admitted patient care with the critical care minimum dataset (CCMD) to obtain details of the intensive therapy unit (ITU) stay. The CCMD is collected from all hospitals and other locations which provide all elements of critical care, to support payment, commissioning and national policy analysis. During each admission, we included total number of ITU stays along with total organ support. We also linked the dataset to Office of National Statistics dataset (ONS) to obtain date of death.

### Ethics approval and consent

The research involved analysis of anonymised data routinely collected in the course of normal care and obtained from NHS Digital. Written informed consent was waived due to the nature of the study and pandemic nature of the disease and the study was approved by Health Research Authority and Health and Care Research Wales (HCRW).

### Definitions

We identified all episodes of confirmed COVID-19 by using validated International Classification of Diseases, Tenth Revision, Clinical Modification (ICD-10-CM) code of U07.1 in any of the 20 diagnoses codes between the period of 1st March 2020 and 31st March 2021, in keeping with the objective of the study.

In the study, potential COVID-19 cases without confirmed diagnosis (U07.2) were not considered. ICD-10 and OPCS-4 codes were used to identify AKI cases, comorbidities and patients with kidney failure on chronic dialysis among 20 diagnosis codes (S1 Table). Up to 20 secondary diagnosis codes were also collected and were used to calculate Charlson’s Comorbidity index (CCI) (S2 Table). The severity of CCI was further classified into mild (CCI scores of 1–2), moderate (CCI scores of 3–4), and severe (CCI scores ≥ 5) [18]. Patients with kidney failure on chronic dialysis were excluded. English regions were extracted from region codes in HES data and patients from non-English regions were excluded. There are nine English regions which were established in 1994 and are the highest tier of sub-national division in England. We defined admission methods as elective, emergency, maternity and child, transfers and unknown. Ethnicity was grouped into six categories as White, Mixed, Asian, Black, other ethnic groups and ethnicity not stated/unknown. English Index of Multiple Deprivation (IMD) deciles were summarised as a categorical factor with decile of 1 indicating that the postcode is in the bottom 10% of the deprivation index, a decile of 2 indicating that the postcode is in the bottom 10–20%, and so on.

We divided the study period according to the predominance of SARS CoV-2 variants and also in relation to the landmark RECOVERY trial. During the first phase from 1st March 2020 to 21st December 2020, “Original or wild type” variant of SARS CoV-2, referred to as “Original” was predominant, while from 22nd December 2020 to 17th May 2021, “Alfa” variant of SARS CoV-2 was the predominant strain [19]. The end date of each phase was chosen based on more than 50% decline in the variant. The RECOVERY trial was published on 22nd June 2020 which led to widespread use of steroids in treatment of COVID-19 [20]. We further defined national patterns of the pandemic by classifying the reported number of cases and deaths in three sequential phases depending on the introduction of steroids as a consequence of the landmark RECOVERY trial. The study period was categorized into three phases: Pre-RECOVERY with the “Original” SARS CoV-2 variant, Post-RECOVERY with the “Original” SARS CoV-2 variant, and post-RECOVERY with the “Alfa” variant of SARS CoV-2.

### Outcome measures

We performed two analyses to understand variation in COVID-19 related outcomes. First, we compared the demographic characteristics of all admitted patients with COVID-19 disease in all nine regions of England, describing the regional epidemiology. Second, we assessed the predictors of death and AKI in COVID-19.

### Statistical analysis

All analysis were performed using IBM SPSS Statistics for Windows, Version 28·0. Armonk, NY: IBM Corp. Characteristics of the study population, categorised as per English regions, were summarised by means and standard deviation for continuous measurements and as percentages for categorical factors. Considering the significant role of population density in the spread of infectious diseases, we acquired population estimates for each region from the Office of National Statistics. Subsequently, we illustrated the regional incidence of hospitalized COVID-19 per million population [21]. We performed descriptive statistical analysis comparing continuous variable using ANOVA and have presented continuous variables as mean with standard deviation (SD). Categorical variables are reported as proportions and percentages and were compared using chi-squared test or Fisher’s exact test. Due to low number of missing data, we did not perform multiple imputation. Data were analysed on each admission of COVID-19 and included patients who had experienced multiple episodes of COVID-19 in separate admission periods. To ensure fairness in the analysis, every admission period was given an equal chance for a binary outcome. This approach avoids survival bias associated with the first admission and mortality bias associated with the last admission.

Multivariable logistic regression models were implemented to identify predictors of in-hospital mortality adjusting for age, gender, ethnicity, admission methods, comorbidity severity, AKI, acute dialysis, intensive care stay, SARS CoV-2 variant, CCI grades, IMD, English regions and the time-period in relation to the publication of COVID variant and RECOVERY trial (RECOVERY phase). In model 2, we excluded comorbidity categories. In model 3, we excluded individual comorbidities. In model 4, we excluded COVID variant, acute dialysis treatment and comorbidity categories. The final model included all demographic variables, admission method, AKI, ITU admission, comorbidity categories, IMD, English region and RECOVERY phase. We present the final model as the model strikes a balance between goodness of fit and simplicity while ensuring its ability to provide meaningful insights into the relationships between variables. The other models are presented in supplementary file. For all models, we determined the odds of death in each region of England with London as the reference.

In keeping with the second objective, we created a multivariable logistic regression model using AKI as outcome and included all demographic variables, admission methods, comorbidity severity, index of multiple deprivation, ITU admission, RECOVERY trial period, and English regions. We also performed sensitivity analysis by including individual comorbidities. Results are presented as odds ratios (ORs) and 95% confidence intervals (CIs). All tests were 2-tailed, and *p* < 0.05 was considered significant.

### Patient and public involvement

Patients were not actively engaged in the creation and execution of the cohort study, the formulation of research questions and objectives, nor in the analysis and documentation of the findings. Due to the study’s inception and execution coinciding with the early stages of the pandemic, involving patients and the public would have presented significant challenges and could have hindered the rapid implementation of the research.

## Results

**Fig. 1:**
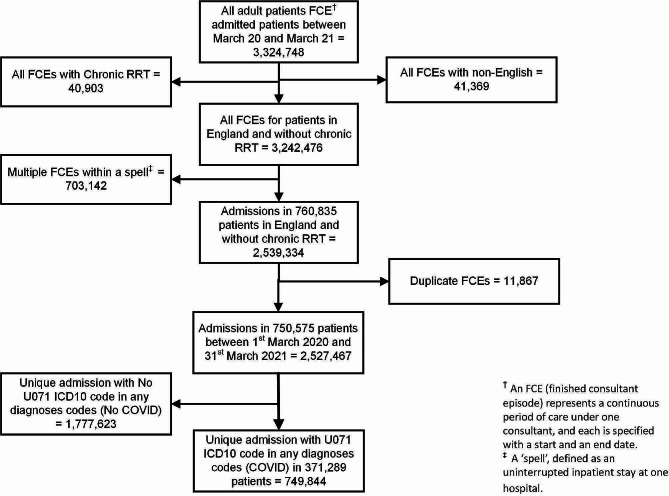
Study flowchart

We extracted 2,539,334 finished consultant episodes (FCEs) for 760,835 patients, residing in England and admitted between 1st March 2020 and 31st March 2021 and who were not receiving chronic dialysis (Fig. 1). After exclusion of 11,867 duplicate FCEs, there were 750,575 patients with 2,527,467 admissions. Of these, there were 749,844 unique admission spells with ICD10 code of U071 in one of the diagnoses codes in 371,289 patients.

### Variation in demographic characteristic in each region

**Fig. 2:**
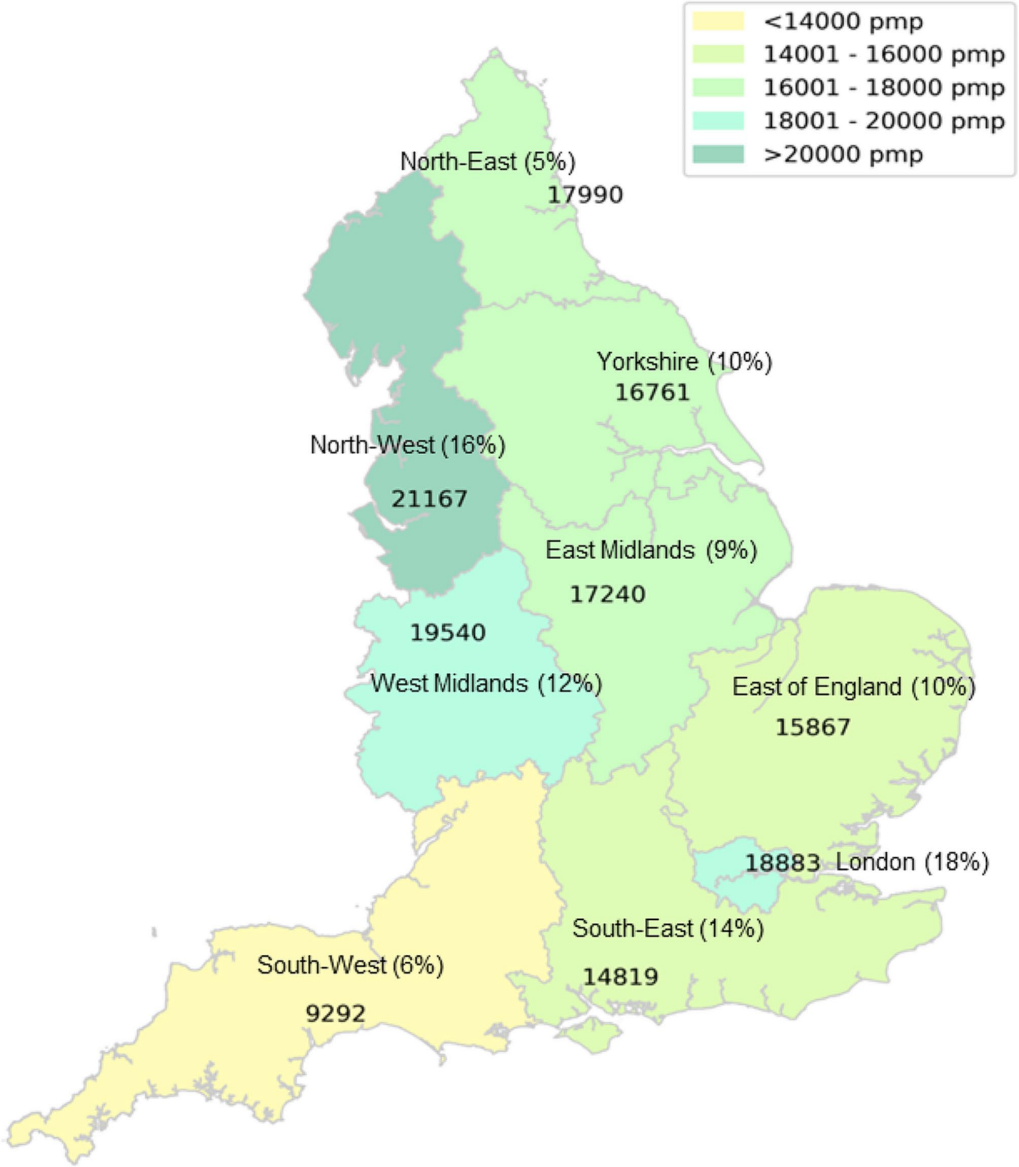
Population incidence of hospitalised COVID-19 in English regions.

Figure 2 and Table 1a shows population incidence and regional proportion of patients with COVID-19. Though London had the highest number of COVID-19 admissions at 131,338 (18% of total in England) followed by the North-West region with 122,683 admissions (16%), the population incidence of COVID-19 hospital admissions was highest in the North West at 21,167 per million population (pmp) and lowest in the South West at 9,292 admission pmp. There was no correlation between population density and population incidence of COVID-19 hospitalization (S1 Fig). In London, patients with COVID19 were younger (67.0 ± 17.7 years) as compared to East of England (72.2 ± 16.8 years). Length of stay was lowest in the North East at 12.2 ± 14.9 days and highest in the North West at 15.2 ± 17.9 days. All regions had male preponderance in COVID-19 hospital admissions. The ethnic composition of COVID-19 cases in London differed from that of other regions, showing greater percentages of individuals from non-white ethnic backgrounds - Asian (16.6%), Black (13%), and other ethnic groups (8.9%). Incidence of AKI varied from 25.8% in the South West to 33.4% in London. Delivery of acute KRT was highest in London at 6.5% and lowest in the North East and Yorkshire at 2.5%.

**Table 1:** **a** Regional demographic characteristics of patients hospitalised with COVID-19; **b** Regional demographic characteristics of patients hospitalised with COVID-19. Values are numbers (%)

		London N (%)	North-East N (%)	North-West N (%)	Yorkshire N (%)	East Midlands N (%)	West Midlands N (%)	East of England N (%)	South-East N (%)	South-West N (%)	Total
Number of admissions		131,338	38,627	122,683	72,942	66,508	90,970	77,953	106,578	42,245	749,844
Population incidence^§^		18,883	17,990	21,167	16,761	17,240	19,540	15,867	14,819	9,292	16,881
Age in years^┼^		67 ± 17.7	71.1 ± 16.4	70.5 ± 16.6	71.2 ± 17.2	71.2 ± 16.9	70.6 ± 17.3	72.2 ± 16.8	71.2 ± 17.1	71 ± 17.8	70.3 ± 17.8
Gender	Male	74,035 (56.4)	20,486 (53.0)	66,391 (54.1)	38,885 (53.3)	36,298 (54.6)	49,086 (54.0)	42,791 (54.9)	58,344 (54.7)	23,224 (55.0)	409,540 (54.6)
Length of stay (days)^┼^		12.5 ± 16.5	12.2 ± 14.9	15.2 ± 17.9	13.4 ± 16.4	14.1 ± 16.6	13.2 ± 14.7	13.3 ± 15.1	13.9 ± 16.3	14.9 ± 17.9	13.7 ± 16.4
Ethnicity ^╪^	White	59,008 (44.9)	34,791 (90.1)	104,653 (85.3)	59,750 (81.9)	52,540 (79.0)	64,586 (71.0)	62,014 (79.6)	82,238 (77.2)	34,383 (81.4)	553,963 (73.9)
	Mixed	1674 (1.3)	104 (0.3)	617 (0.5)	387 (0.5)	401 (0.6)	639 (0.7)	512 (0.7)	841 (0.8)	260 (0.6)	5435 (0.7)
	Asian	21,759 (16.6)	725 (1.9)	6658 (5.4)	5613 (7.7)	5020 (7.6)	10,021 (11.0)	3621 (4.7)	5450 (5.1)	863 (2.0)	59,730 (8.0)
	Black	17,112 (13.0)	135 (0.4)	1767 (1.4)	989 (1.4)	1254 (1.9)	3232 (3.6)	1598 (2.1)	1481 (1.4)	443 (1.1)	28,011 (3.7)
	“Other”	11,683 (8.9)	367 (1.0)	1718 (1.4)	965 (1.3)	972 (1.5)	1361 (1.5)	1274 (1.6)	2551 (2.4)	443 (1.1)	21,334 (2.9)
	Not known	20,102 (15.3)	2505 (6.5)	7270 (5.9)	5238 (7.2)	6321 (9.5)	11,131 (12.2)	8934 (11.5)	14,017 (13.2)	5853 (13.9)	81,371 (10.9)
Admission method ^╪^	Elective	2107 (1.6)	498 (1.3)	2139 (1.8)	1180 (1.7)	1013 (1.7)	1054 (1.2)	1275 (1.7)	1711 (1.7)	1050 (2.6)	12,027 (1.7)
	Emergency	120,470 (93.5)	34,958 (92.7)	114,292 (94.2)	67,706 (95.1)	56,986 (95.0)	83,646 (94.3)	71,492 (93.7)	97,490 (94.8)	36,266 (90.9)	683,306 (94.0)
	Maternity and Child	2116 (1.6)	310 (0.8)	1335 (1.1)	921 (1.3)	658 (1.1)	1278 (1.4)	744 (1.0)	946 (0.9)	369 (0.9)	8,677 (1.2)
	Transfers	4214 (3.3)	1932 (5.1)	3605 (3.0)	1415 (2.0)	1339 (2.2)	2673 (3.0)	2749 (3.6)	2686 (2.6)	2206 (5.5)	22,819 (3.1)
AKI^╪^		43,924 (33.4)	11,951 (30.9)	35,008 (28.5)	21,459 (29.4)	19,870 (29.9)	27,262 (30.0)	24,560 (31.5)	32,352 (30.4)	10,882 (25.8)	227,268 (30.3)
Acute RRT^╪^		2870 (6.5)	301 (2.5)	1356 (3.9)	532 (2.5)	577 (2.9)	1238 (4.5)	873 (3.6)	1167 (3.6)	365 (3.4)	9279 (4.1)
Charlson comorbidity index (CCI) grades	No comorbidity	38,201 (29.1)	8707 (22.5)	27,237 (22.2)	16,192 (22.2)	14,770 (22.2)	19,802 (21.8)	18,636 (23.9)	26,500 (24.9)	10,937 (25.9)	180,982 (24.1)
	Mild comorbidities	54,753 (41.7)	16,293 (42.2)	52,115 (42.5)	30,646 (42.0)	26,562 (39.9)	38,103 (41.9)	34,797 (44.6)	44,122 (41.4)	17,794 (42.1)	315,185 (42.0)
	Moderate comorbidities	25,352 (19.3)	9125 (23.6)	28,433 (23.2)	16,971 (23.3)	16,313 (24.5)	20,984 (23.1)	16,965 (21.8)	23,538 (22.1)	9192 (21.8)	166,873 (22.3)
	Severe comorbidities	13,032 (9.9)	4502 (11.7)	14,898 (12.1)	9133 (12.5)	8863 (13.3)	12,081 (13.3)	7555 (9.7)	12,418 (11.7)	4322 (10.2)	86,804 (11.6)
Deprivation National Deciles *	10	2813 (2.1)	1191 (3.1)	6040 (4.9)	2963 (4.1)	5181 (7.8)	4633 (5.1)	8966 (11.5)	17,635 (16.6)	3703 (8.8)	53,125 (7.1)
	9	6118 (4.7)	2062 (5.3)	8672 (7.1)	4436 (6.1)	6724 (10.1)	4578 (5.0)	8948 (11.5)	13,900 (13.0)	4417 (10.5)	59,855 (8.0)
	8	7657 (5.8)	2164 (5.6)	8892 (7.3)	5424 (7.4)	5999 (9.0)	7088 (7.8)	9346 (12.0)	11,609 (10.9)	4591 (10.9)	62,770 (8.4)
	7	8765 (6.7)	2468 (6.4)	9236 (7.5)	6118 (8.4)	6578 (9.9)	7159 (7.9)	9043 (11.6)	11,313 (10.6)	5008 (11.9)	65,688 (8.8)
	6	11,548 (8.8)	2384 (6.2)	9549 (7.8)	6666 (9.1)	6150 (9.3)	7527 (8.3)	9363 (12.0)	12,075 (11.3)	5684 (13.5)	70,946 (9.5)
	5	26,610 (20.3)	6931 (17.9)	16,542 (13.5)	9437 (12.9)	7153 (10.8)	13,671 (15.0)	4871 (6.3)	6988 (6.6)	2742 (6.5)	95,955 (12.7)
	4	23,162 (17.6)	5435 (14.1)	13,029 (10.6)	8140 (11.2)	8352 (12.6)	10,162 (11.2)	7071 (9.1)	8480 (8.0)	3551 (8.4)	87,382 (11.7)
	3	17,363 (13.2)	4353 (11.3)	11,410 (9.3)	8018 (11.0)	7032 (10.6)	7843 (8.6)	8717 (11.2)	10,345 (9.7)	4795 (11.4)	79,876 (10.7)
	2	13,108 (10.0)	3573 (9.3)	9637 (7.9)	6053 (8.3)	6997 (10.5)	8672 (9.5)	8713 (11.2)	10,888 (10.2)	5490 (13.0)	73,131 (9.8)
	1	14,194 (10.8)	8066 (20.9)	29,676 (24.2)	15,687 (21.5)	6342 (9.5)	19,637 (21.6)	2915 (3.7)	3345 (3.1)	2264 (5.4)	102,126 (13.6)
Deaths		19,495 (14.8)	5943 (15.4)	21,075 (17.2)	12,140 (16.6)	11,013 (16.6)	15,039 (16.5)	14,195 (18.2)	17,520 (16.4)	6097 (14.4)	122,517 (16.3)

┼Mean ± standard deviation, § per million population (pmp), ╪ Number (%)

^*^10 = least deprived

**Fig. 3:**
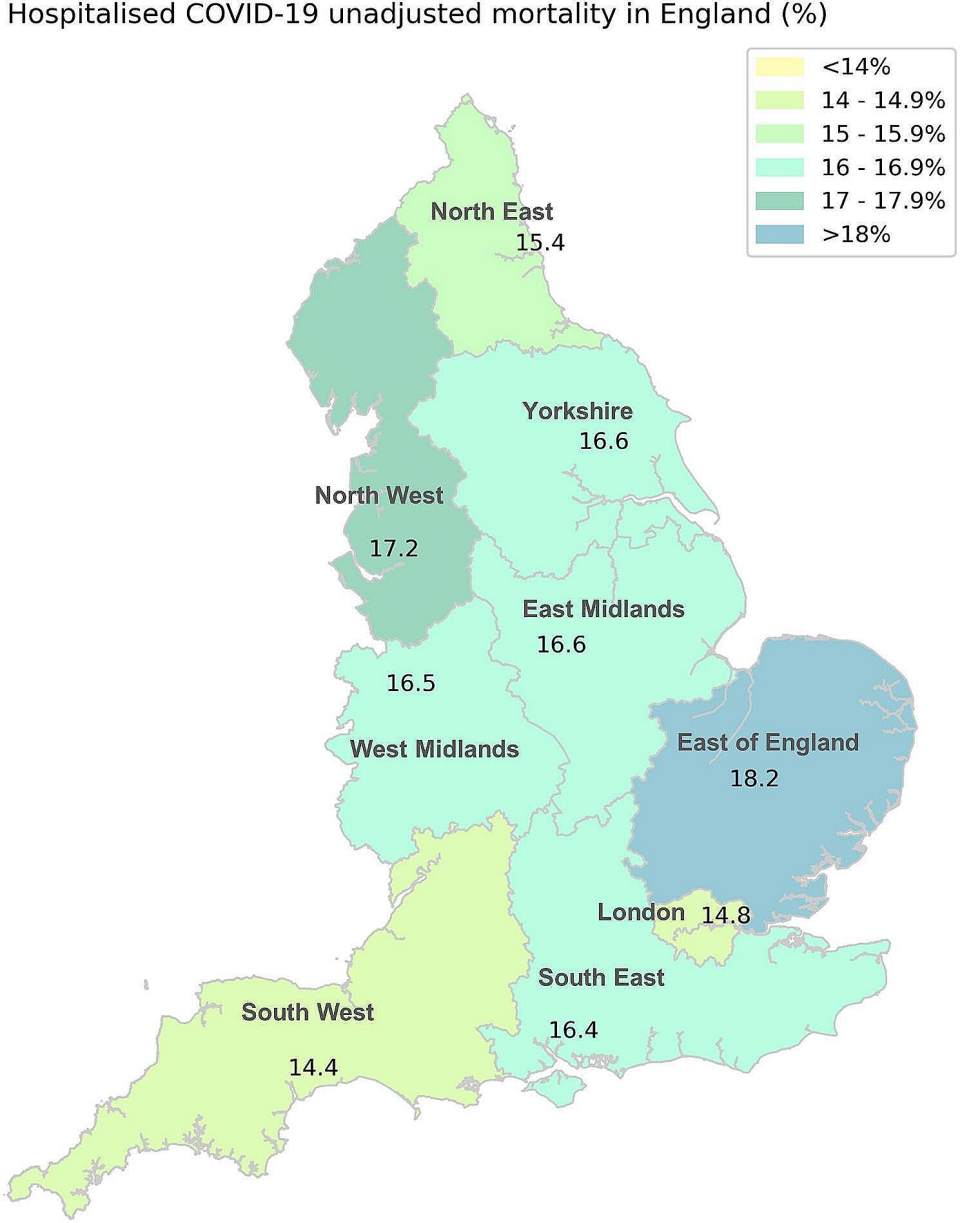
Unadjusted mortality of hospitalised COVID-19 in English regions

The North East, North West, Yorkshire and West Midlands had more than 20% of COVID-19 cases from most deprived areas as compared to East of England (3.7%) and the South East (3.1%) (Table 2b).

There was considerable variation in total comorbidity burden in COVID-19 cases in all regions of England ranging from 39.9 to 44.6% for mild comorbidities, 19.3 to 24.5% for moderate comorbidities and 9.7–12.5% for severe comorbidities. Major comorbidities in those with COVID-19 in all regions were diabetes with or without complications (25.9 to 33%), COPD (25 to 30.3%) and chronic kidney disease (18.9 to 24.4%) (S3 Table).

### ITU and COVID-19 hospital admissions

ITU admissions for patients with COVID-19 varied from 5.2% in the North East to 10.3% in London (Table 3). In every region, COVID-19 patients, on average, experienced more than one admission to the ITU throughout the study duration. There was significant variation in the need and duration for organ support in various English regions. Amongst all regions, duration of kidney support was highest in London at 2.7 ± 7.9 days and lowest in the North East at 1.4 ± 4.6 days. Length of stay (LOS) in ITU was highest in London (10.7 ± 15.4 days) and lowest in the North East (6.5 ± 10.3 days).

**Table 2:** ITU characteristics of patients hospitalised with COVID-19 in English regions

	London	North-East	North-West	Yorkshire	East Midlands	West Midlands	East of England	South-East	South-West	Total
Admissions to ITU^┼^	13,505 (10.3)	2010 (5.2)	7944 (6.5)	3966 (5.4)	3884 (5.8)	6200 (6.8)	5611 (7.2)	6719 (6.3)	2517 (6.0)	52,356 (7)
Number of ITU admissions ^╪^	1.2 ± 0.5	1.1 ± 0.3	1.1 ± 0.3	1.1 ± 0.5	1.2 ± 0.6	1.1 ± 0.4	1.1 ± 0.4	1.1 ± 0.5	1.1 ± 0.4	1.1 ± 0.5
Number of organ support required ^╪^	2.6 ± 1.8	2.4 ± 1.4	2.2 ± 1.3	2.4 ± 1.5	2.4 ± 1.6	2.7 ± 1.6	2.2 ± 1.4	2.3 ± 1.6	2.2 ± 1.7	2.4 ± 1.6
ITU length of stay (days)^╪^	10.7 ± 15.4	6.5 ± 10.3	7.6 ± 12.5	7.1 ± 11.8	8.2 ± 12.1	9.3 ± 11.3	7.4 ± 12.8	9.1 ± 13.4	7.3 ± 12.3	8.7 ± 13.2
Kidney support days^╪^	2.7 ± 7.9	1.4 ± 4.6	1.5 ± 4.9	1.5 ± 4.7	1.5 ± 5.1	1.9 ± 5.3	1.6 ± 5.7	1.8 ± 5.6	1.7 ± 5.7	1.9 ± 6.1
ITU mortality ^┼^	3747 (33.4)	619 (31.0)	1217 (39.5)	1420 (41.7)	684 (39.9)	1794 (37.7)	1542 (34.4)	1569 (32.2)	505 (25.0)	13,097 (34.9)

^┼^ Number (%), ^╪^ mean 2 standard deviations

### Mortality in COVID-19

Unadjusted in-hospital mortality rate in all English regions varied between 14.4% and 18.2%, with highest mortality rate in East of England at 18.2% and lowest in the South West at 14.4% (Table 3; Fig. 3). Unadjusted ITU mortality was highest in Yorkshire at 41.7% and lowest in the North East at 31%. Prior to the RECOVERY trial, unadjusted in-hospital mortality rate was high during the prevalence of “Original” strain of SARS CoV-2 in all regions, highest in East of England at 22.2%. Unadjusted in-hospital mortality rate decreased in all regions after publications of the RECOVERY trial during the periods when “Original” and “Alfa” strains of SARS CoV-2 were dominant. The largest decline in unadjusted in-hospital mortality was observed in Yorkshire (7.3% decline, from 21.6 to 14.3%) and lowest decline was in the South East (4.5% decline, from 19.6 to 15.1%).

**Table 3:** Unadjusted In-hospital mortality in English regions in relation to COVID variant and RECOVERY Trial^†^

	London	North-East	North-West	Yorkshire	East Midlands	West Midlands	East of England	South-East	South-West	Total
Overall unadjusted mortality	19,495 (14.8)	5943 (15.4)	21,075 (17.2)	12,140 (16.6)	11,013 (16.6)	15,039 (16.5)	14,195 (18.2)	17,520 (16.4)	6097 (14.4)	122,517 (16.3)
Pre-RECOVERY “Original” SARS CoV-2 variant	7272 (19.6)	1771 (19.4)	6525 (21.1)	3540 (21.6)	3039 (19.9)	4609 (20.7)	4299 (22.2)	4659 (19.6)	1552 (18)	37,266 (20.4)
Post-RECOVERY “Original” SARS CoV-2 variant	3844 (13)	2177 (14.4)	8001 (16.6)	4994 (15.9)	3698 (16)	4520 (15.5)	3274 (17.9)	4476 (16.4)	1913 (14.3)	36,897 (15.6)
Post-RECOVERY “Alfa” SARS CoV-2 variant	8379 (12.9)	1995 (13.8)	6549 (15)	3606 (14.3)	4276 (15.2)	5910 (15)	6622 (16.4)	8385 (15.1)	2632 (13)	48,354 (14.6)

^┼^ Numbers (%)

### Predictors of mortality

**Fig. 4:**
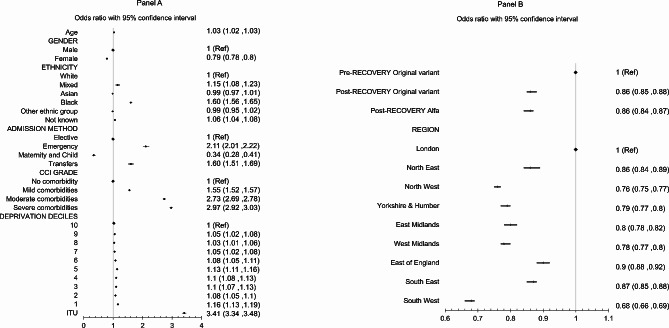
Multivariate analysis of predictors of mortality in patients hospitalised with COVID-19

In multivariable logistic regression analysis, age (OR 1.05, 95% CI 1.04, 1.05 per year), Asian ethnicity (OR 1.07, 95% CI 1.04, 1.10), emergency admission (OR 1.83, 95% CI 1.71, 1.96), transfers from other hospitals (OR 1.49, 95% CI 1.38, 1.62), presence of AKI (OR 2.26, 95% CI 2.23, 2.29) and ITU admission (OR 7.18, 95% CI 7.02, 7.35) were associated with higher odds of death (Fig. 4 panel A). As compared to least deprived, increasing indices of deprivation were associated with higher odds of death, though this was not incremental (Fig. 4 Panel B). When compared with no comorbidity, higher comorbidity categories were associated with higher odds of death - mild (OR 1.60, 95% CI 1.56, 1.63), moderate (OR 1.05, 95% CI 1.04, 1.05) and severe comorbidities (OR 2.64, 95% CI 2.58, 2.71).

As compared to London, all eight other regions had higher odds of death, ranging from OR of 1.04, 95% CI 1.00, 1.07) in the South West to OR 1.24 (95% CI 1.21, 1.28) in the North West (Fig. 4 Panel C). Odds of death were lower in patients with COVID-19 in the post RECOVERY period, both with the “Original” (OR 0.72, 95% CI 0.71, 0.74) and “Alfa” strain of SARS CoV-2 (OR 0.75, 95% CI 0.74, 0.76).

### Predictors of AKI

**Fig. 5:**
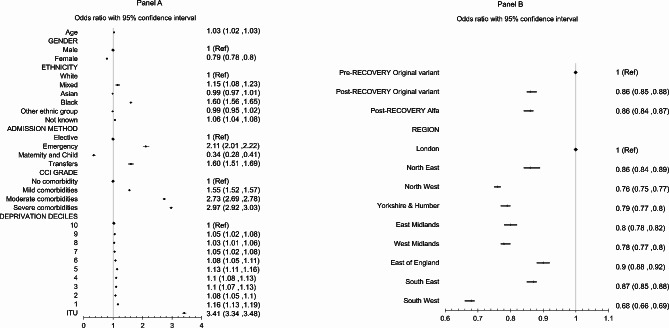
Multivariate analysis of predictors of AKI in patients hospitalised with COVID-19

In multivariable adjusted analysis, increasing age (OR 1.03, 95% CI 1.02, 1.03 per year), mixed ethnicity (OR 1.15, 95% CI 1.08, 1.23), black ethnicity (OR 1.60, 95% CI 1.56, 1.65), emergency admission (OR 2.11, 95% CI 2.01, 2.22), transfers from other provider (OR 1.60, 95% CI 1.51, 1.69), ITU care (OR 3.41, 95% CI 3.34, 3.48), mild comorbidities (OR 1.55, 95% CI 1.52, 1.57), moderate comorbidities (OR 2.73, 95% CI 2.69, 2.78), and severe comorbidities (OR 2.97, 95% CI 2.92, 3.03) were associated with higher odds of developing AKI (Fig. 5 Panel A). Higher indices of deprivation were also independently associated with increasing odds of developing AKI, though there was no incremental trend with increasing deprivation. Female gender (OR 0.79, 95% CI 0.78, 0.80) and maternity admissions (OR 0.34, 95% CI 0.28, 0.41) had lower odds of developing AKI. Post the RECOVERY publication, the “Original” variant (OR 0.87, 95% CI 0.85, 0.88) and “Alfa variant (OR 0.87, 95% CI 0.86, 0.88) had similar and lower odds of developing AKI (Fig. 5 Panel B). All eight regions in England had lower odds of developing AKI as compared to London.

### Sensitivity analysis

We performed various sensitivity analysis by initially including individual comorbidities, acute dialysis and COVID variants to other variables and then removing each of them to give us the final model. The results of sensitivity analysis confirmed the primary results (S4 Table). We also performed sensitivity analysis for predictors of AKI by including individual comorbidities which confirmed our findings (S5 Table).

## Discussion

In this large national study of hospital admissions, we found large regional differences in COVID-19 hospitalization with the highest population incidence of hospitalization in the North West region of England and lowest in the South West region. Though London evidenced considerable ethnic diversity with a high proportion of Asian and Black patients and lower mean age at hospitalization, the population incidence of admissions was lower than in the North West and West Midlands regions. Unexpectedly, we found poor correlation between population density and incidence of hospitalization. The common perception is that higher population density increases social contacts resulting in increased transmission of infection and resultant adverse events like hospitalization. Nevertheless, while some studies have a found a positive correlation between COVID-19 hospitalisation and population density others have found that population density was not an important factor in COVID-19 spread [3, 22]. Infection control measures like social distancing, mask wearing and contact tracing which were introduced in England, may have impacted the relationship between population density and infection rates [23]. The association with social deprivation suggests that poor housing and inability to comply with restrictions as well as lack of availability of hospital beds may have been a factor contributing to decreased hospitalization rates in some regions [24]. In addition, some studies have suggested that the observed differences in hospitalization could potentially indicate variations in the quality of community care provided but acknowledge that some of these differences might also be attributed to variances in hospital admission criteria [25, 26].

In our study, we found that patients admitted to hospitals in London were predominantly from non-white ethnic background, younger, had higher proportion with AKI and needing acute KRT as compared to all other regions in England. London also had a higher proportion of admissions to ITU. Despite this, London unexpectedly evidenced the lowest adjusted odds of death as compared to all other regions. Our study results are in keeping with a study performed during the first five months of the pandemic, to evaluate regional variation in COVID-19 mortality [16]. The authors used the HES dataset and included only the last admission of patients with confirmed and possible diagnosis of COVID as identified by ICD10 codes of U071 and U072. The regional variation in in-hospital mortality, though higher, was similar to our study with highest in East of England (29.9%) and lower mortality in the South West (22.7%). They also found that Trusts with higher ITU admissions had lower adjusted in-hospital mortality.

One important consideration in interpreting these data is that COVID-19 leads to notably higher death rates among elderly individuals, who are often located in regions with a lower ratio of hospital beds to the population. Although London has the fewest beds per person across all age groups, its relatively smaller population of older residents allows for a relatively greater availability of beds for the older population who may require them the most [27]. The East Midlands region in England has the lowest number of hospital beds relative to the population of older people with only 102.2 beds per 10,000 people aged 70 years or older compared with the highest rate of 165.5 beds per 10,000 people aged 70 years or older in London. In terms of ITU beds per capita, London has 30% more beds compared to the South West region [28]. Though this study was not designed to provide in-depth analysis of the reasons for regional variation in mortality, the unexplained variation should stimulate discussion and consideration of the reasons for lower mortality in London despite higher rates of AKI and KRT. The availability of hospital and ITU beds as well as the speed of adoption of best practice may all have been factors that are relevant to consider in preparation for future pandemics.

We found lower odds of death for COVID-19 admissions with black ethnicity and higher odds of death with Asian ethnicity. In contrast, many studies have found higher mortality in Black and Asian patients hospitalised with COVID-19 [29, 30]. Similarly, a recent study of community-based data of COVID-19 hospitalization using the OpenSafely platform, covered two waves of the COVID pandemic from February 2020 to August 2020 and September to December 2020 and found lower odds of hospitalization and mortality in patients with white ethnicity as compared to all other ethnicities [31]. Our data therefore confirm the higher risk of death associated with COVID-19 in Asian patients. The lower observed risk of death in Black patients is unexplained and warrants further investigation, though lower odds of death have been reported in the black Caribbean population in the second phase of the pandemic [17].

The London region also had a higher incidence of AKI associated with COVID-19 and greater proportion of patient’s needing acute KRT. All regions in England had lower adjusted risk of developing AKI as compared to London. The elevated likelihood of AKI and increased occurrence of KRT in AKI cases in London, aligns with similar findings in patients in England without COVID-19 [32]. The higher probability of AKI occurrence in London might be linked to the larger representation of males and individuals from ethnic minority backgrounds in its population. Both of these factors have been connected to an elevated risk of developing AKI [33, 34]. The lower mortality rate observed in London can be attributed to the presence of a younger population and a diverse ethnic composition. Previous studies conducted in England have indicated a reduced mortality rate among individuals with Black and Asian ethnicity in both non-dialysis and dialysis requiring AKI [35, 36]. Vaccination uptake in the population may also have a role in preventing AKI. United Kingdom government policy rightly prioritised the older population for vaccination. Vaccination distribution varied between regions in England and London, with its younger population, had lower vaccination rates [37].

Our results show a decrease in mortality after the publication of the RECOVERY trial which may have led to rapid adoption of steroid treatment in severe COVID-19. The decline in mortality was evident during the predominant prevalence of both “Original” and “Alfa” strain of SARS CoV-2 and is in keeping with various trials and a metanalysis of steroid use in COVID-19 [38, 39]. In another study of suspected and confirmed COVID admissions in England between March and September 2020, the authors found a substantial decline in mortality between March to June 2020 (pre-RECOVERY) and July to September 2020 (post-RECOVERY) [4].

However, this study has limitations, such as including only patients with confirmed COVID-19 and admitted to the hospital, potentially excluding patients with suspected COVID-19 in the early stages of the pandemic when testing was limited. The study uses ICD-10 codes to identify COVID-19 and AKI which has its own limitations, such as lack of specificity and regional variation in coding practice, however AKI, specifically, presents an excellent opportunity for research utilizing administrative health data because it is highly common among hospitalized individuals [40]. NHS Digital has embedded quality assurance checks within the design of the Secondary User Services and HES dataset to minimize the variation in coding practices and ensure completion of data items. Comprehensive assessments of data accuracy in UK health records have revealed both deficiencies and advancements in discharge coding precision from 2001 to 2011. The 2011 review demonstrated that, subsequent to improvements associated with Payment by Results (PbR), primary diagnosis accuracy increased significantly from 74% (interquartile range [IQR] 59–92%) to 96% (89–96%, *p* = 0.02) [41, 42]. Though regional variation in coding practices cannot be ruled out, our study was during the recent pandemic when there was extreme focus of COVID-19, which may have ensured appropriate coding. However, inspite of this, clinical variables like blood pressure, body weight, and blood results that could influence hospitalization and outcomes were not available. Thirdly, HES dataset lacks information on several social factors, such as housing, smoking, employment and vaccination. Thirdly, HES dataset lacks information on several social factors, such as housing, smoking, employment and vaccination. Though, many of these factors were covered in the IMD, which we categorized, we didn’t have data for vaccination. The greatest strength of this study is its reliance on a comprehensive national database that includes all admissions in the NHS, with strong connections to the ITU database and ONS dataset to ensure all confirmed COVID-19 cases were accounted for. Our research contributes to existing knowledge on hospitalizations and mortality during the COVID-19 pandemic in England by presenting in-depth data on regional differences over 13 months, including information on AKI, a significant COVID-19 complication and risk factor for mortality [17].

## Conclusions

Our research sheds light on unexplained differences in hospitalization, AKI and mortality rates observed in regions across England during the COVID-19 pandemic. This is particularly notable, given the existence of a single health care system, the National Health Service. The variability in mortality and AKI rates within regions and their correlation with important risk factors should be taken into consideration in preparation for future pandemics in England and globally.

### Electronic supplementary material

Below is the link to the electronic supplementary material.

Supplementary Material 1

## Data Availability

The authors are unable to fulfil requests for underlying data due to the data sharing agreement with NHS Digital, which governs the acquisition and analysis of HES data. Access to HES data can be obtained by directly applying to NHS Digital, subject to their conditions of use and further usage policies.
