# Supplementary material for: Regional variation of COVID-19 admissions, acute kidney injury and mortality in England - a national observational study using administrative data
Source: BMC Infect Dis. 2024 Mar 22;24:346. doi: 10.1186/s12879-024-09210-6 (PMC10960376; doi:10.1186/s12879-024-09210-6)
Supplement: Supplementary file 1 — Supplementary Material 1 [file 12879_2024_9210_MOESM1_ESM.docx]

**Supplementary material**

**Regional variation of COVID-19 admissions, acute kidney injury and mortality in England - a national observational study using administrative data.**

Nitin V Kolhe^1,2^, Richard J Fluck^1,2^, Maarten W Taal^1,2^

^1^ University Hospitals of Derby and Burton NHS Trust, ^2^ Centre for Kidney Research and Innovation, Academic Unit for Translational Medical Sciences, School of Medicine, University of Nottingham, Nottingham, UK.

Table of contents

| S1 Table | International classification of disease 10^th^ edition (ICD-10) and Office of Population Censuses and Surveys Classification of Interventions and Procedures, 4th revision (OPCS-4) codes used in the study. | 2 |
| --- | --- | --- |
| S2 Table | ICD-10 codes used in Charlson's comorbidity score. | 3 |
| S3 Table | Comorbidities in patients hospitalised with COVID-19. | 4 |
| S4 Table | Other ITU characteristics of patients hospitalised with COVID-19 in English regions. | 5 |
| S5 Table | Sensitivity analysis for mortality predictors in patients hospitalised with COVID-19 | 6-8 |
| S6 Table | Sensitivity analysis for AKI predictors in patients hospitalised with COVID-19 | 9-10 |
| S1 Figure | Correlation between population density and population incidence of hospitalised COVID-19. | 11 |
| S7 Table | STROBE Statement | 12-15 |
| S1 Text | Protocol | 16 |

S1 Table: International classification of disease 10^th^ edition (ICD-10) and Office of Population Censuses and Surveys Classification of Interventions and Procedures, 4th revision (OPCS-4) codes used in the study.

| **COVID-19** | |
| --- | --- |
| U07.1 | COVID-19 virus identified |
| U07.2 | COVID-19 virus not identified |
| **AKI** | |
| N17.0 | Acute renal failure with tubular necrosis |
| N17.1 | Acute renal failure with acute cortical necrosis |
| N17.2 | Acute renal failure with medullary necrosis |
| N17.8 | Other acute renal failure |
| N17.9 | Acute renal failure, unspecified |
|  |  |
| **Dialysis codes** | |
| X40.2 | Peritoneal dialysis |
| X40.3 | haemodialysis |
| X40.4 | haemofiltration |
| X40.5 | Automated peritoneal dialysis |
| X40.6 | Continuous ambulatory peritoneal dialysis |
| Z99.2 | Dependence on renal dialysis |

S2 Table: ICD-10 codes used in Charlson's comorbidity score.

| **Comorbidities** | **ICD-10 *** |
| --- | --- |
| Myocardial infarction (MI) | I21x, I22x, I252 |
| Congestive heart failure (CCF) | I099,I110, I130, I132, I255, I420, |
|  | I425-I429, I43x, I50x, P290 |
|  |  |
| Peripheral vascular  Disease (PVD) | I70x, I71x, I731, I738, I739, I771, |
|  | I790, I792, K551, K558, K559, |
|  | Z958, Z959 |
| Cerebrovascular disease (CVD) | G45x, G46x, H340, I60x-I69x |
| Dementia | F00x-F03x, F051, G30x, G311 |
| Chronic obstructive pulmonary disease (COPD) | I278, I279, J40x-J47x, J60x-J67x, |
|  | J684, J701, J703 |
| Connective tissue disorder (CTD) | M05x, M06x, M315, M32x-M34x, |
|  | M351, M353, M360 |
| Peptic ulcer disease (PUD) | K25x-K28x |
| Mild liver disease | B18x, K700-K703, K709, |
|  | K713-K715, K717, K73x, K74x, |
|  | K760, K762-K764, K768, K769, |
|  | Z944 |
| Diabetes without chronic  complication | E100, E10l, E106, E108, E109, |
|  | E110, E111, E116, E118, E119, |
|  | E120, E121, E126, E128, E129, |
|  | E130, E131, E136, E138, E139, |
|  | E140, E141, E146, E148, E149 |
| Diabetes with chronic  complication | E102-E105, E107, E112-E115, |
|  | E117, E122-E125, E127, |
|  | E132-E135, E137, E142-E145, |
|  | E147 |
| Hemiplegia or paraplegia | G041, G114, G801, G802, G81x, |
|  | G82x, G830-G834, G839 |
| Renal disease (CKD) | I120, I131, N032-N037, |
|  | N052-N057, N18x, N19x, N250, |
|  | Z490-Z492, Z940, Z992 |
|  |  |
| Any malignancy,  including lymphoma  and leukemia, except  malignant neoplasm of skin | C00x-C26x, C30x-C34x, |
|  | C37x-C41x, C43x, C45x-C58x, |
|  | C60x-C76x, C81x-C85x, C88x, |
|  | C90x-C97x |
|  |  |
| Moderate or severe liver  disease | I850, I859, I864, I982, K704, K711, |
|  | K721, K729, K765, K766, |
|  | K767 |
| Metastatic solid tumor | C77x-C80x |
| AIDS/HIV | B20x-B22x, B24x |

S3 Table: Comorbidities in patients hospitalised with COVID-19.

|  |  | London | North-East | North-West | Yorkshire | East Midlands | West Midlands | East of England | South-East | South-West | Total |
| --- | --- | --- | --- | --- | --- | --- | --- | --- | --- | --- | --- |
| Comorbidities | MI | 10458 (8.0) | 3706 (9.6) | 13336 (10.9) | 8464 (11.6) | 6911 (10.4) | 9373 (10.3) | 7218 (9.3) | 10573 (9.9) | 4045 (9.6) | 74,084 (9.9) |
|  | CCF | 15974 (12.2) | 5089 (13.2) | 19878 (16.2) | 12681 (17.4) | 13098 (19.7) | 16342 (18.0) | 11793 (15.1) | 17007 (16.0) | 6091 (14.4) | 117,953 (15.7) |
|  | PVD | 5213 (4.0) | 2526 (6.5) | 7975 (6.5) | 4878 (6.7) | 3734 (5.6) | 4856 (5.3) | 3919(5.0) | 6026 (5.7) | 2299 (5.4) | 41,426 (5.5) |
|  | CVD | 12609 (9.6) | 2809 (7.3) | 11596 (9.5) | 6667(9.1) | 6768 (10.2) | 10551 (11.6) | 7542 (9.7) | 14176 (13.3) | 3961 (9.4) | 76,679 (10.2) |
|  | Dementia | 14817 (11.3) | 5294 (13.7) | 17218 (14.0) | 10795 (14.8) | 10642(16.0) | 12967 (14.3) | 12731 (16.3) | 14849 (13.9) | 5518 (13.1) | 104,831 (14.0) |
|  | COPD | 29346 (22.3) | 11695 (30.3) | 35961 (29.3) | 21475 (29.4) | 18298 (27.5) | 25634 (28.2) | 19479 (25.0) | 27494 (25.8) | 10540 (25.0) | 199,922 (26.7) |
|  | CTD | 4101 (3.1) | 1631 (4.2) | 4706 (3.8) | 3380 (4.6) | 3396 (5.1) | 4515 (5.0) | 3547 (4.6) | 4643 (4.4) | 1937 (4.6) | 31,856 (4.3) |
|  | Hemi/Paraplegia | 3345 (2.6) | 976 (2.5) | 2733 (2.2) | 1633 (2.2) | 1664 (2.5) | 2028 (2.2) | 1544 (2.0) | 2291 (2.2) | 504 (1.2) | 16,718 (2.2) |
|  | PUD | 861 (0.7) | 408 (1.1) | 1082 (0.9) | 665 (0.9) | 421 (0.6) | 775 (0.9) | 628 (0.8) | 821 (0.8) | 326 (0.8) | 5,987 (0.8) |
|  | Kidney disease | 24801 (18.9) | 9249 (23.9) | 27084 (22.1) | 16841 (23.1) | 16250 (24.4) | 21397 (23.5) | 15082 (19.4) | 22398 (21.0) | 9599 (22.7) | 162,701 (21.7) |
|  | Diabetes | 43333 (33.0) | 10885 (28.2) | 34432 (28.1) | 21868 (30.0) | 19966 (30.0) | 29023 (31.9) | 21552 (27.7) | 29435 (27.6) | 10956 (25.9) | 221,450 (29.5) |
|  | Liver disease | 6806 (5.2) | 2107 (5.5) | 6501(5.3) | 3245 (4.5) | 2706 (4.1) | 3842 (4.2) | 2631 (3.4) | 4986 (4.7) | 1794 (4.3) | 34,618 (4.6) |
|  | Cancer | 9472 (7.2) | 3735 (9.7) | 11488 (9.4) | 6314 (8.7) | 6391 (9.6) | 8502 (9.4) | 7225 (9.3) | 9672 (9.1) | 3799 (9.0) | 66,598 (8.9) |

S4 Table: Other ITU characteristics of patients hospitalised with COVID-19 in English regions.

|  | London | North-East | North-West | Yorkshire | East Midlands | West Midlands | East of England | South-East | South-West | Total |
| --- | --- | --- | --- | --- | --- | --- | --- | --- | --- | --- |
| Total advanced cardiac support days ^‡^ | 1.6 ± 4.8 | 0.6 ± 2.4 | 0.6 ± 2 | 1.1 ± 2.9 | 1.1 ± 2.9 | 1.5 ± 3.6 | 1.2 ± 3.6 | 1.6 ± 45.6 | 0.9 ± 2.9 | 1.2 ± 16.3 |
| Total advanced respiratory support days^‡^ | 10.4 ± 14.8 | 6.3 ± 10.2 | 7.7 ± 12.5 | 6.9 ± 11.7 | 7.8 ± 11.8 | 9.1 ± 11.2 | 7.2 ± 12.5 | 9.4 ± 48.8 | 7 ± 12 | 8.6 ± 20.9 |
| Total basic cardiac support days ^‡^ | 11.4 ± 14.2 | 9.1 ± 10.2 | 10.6 ± 11.9 | 9.5 ± 11 | 9.7 ± 11.4 | 10.2 ± 10.7 | 8.1 ± 11 | 10.8 ± 48.5 | 9 ± 11.7 | 10.2 ± 20.4 |
| Total basic respiratory support days^‡^ | 2.8 ± 4.4 | 4.6 ± 4.6 | 3.8 ± 4.7 | 4.7 ± 5.1 | 3.4 ± 4.1 | 3.3 ± 4.4 | 3.6 ± 4.3 | 4.4 ± 47.4 | 4.1 ± 5 | 3.6 ± 17.1 |
| High dependency unit length of stay in days ^‡^ | 3.3 ± 4.5 | 4.9 ± 4.8 | 4.6 ± 5.4 | 5.2 ± 5.2 | 3.7 ± 4.2 | 3.7 ± 4.6 | 3.4 ± 4.2 | 3.7 ± 4.7 | 4.8 ± 5.1 | 3.9 ± 4.8 |

^Ɨ^ Number (%), ^‡^ mean 2 standard deviations.

S5 Table: Sensitivity analysis for mortality predictors in patients hospitalised with COVID-19

|  |  | Model 1 | Model 2 | Model 3 | Model 4 |
| --- | --- | --- | --- | --- | --- |
| Age |  | 1.05 (1.05-1.05) | 1.05 (1.05-1.05) | 1.05 (1.05-1.05) | 1.05 (1.05-1.05) |
| Gender | Male | 1 (Ref) | 1 (Ref) | 1 (Ref) | 1 (Ref) |
|  | Female | 0.79 (0.78-0.8) | 0.8 (0.79-0.81) | 0.79 (0.78-0.8) | 0.79 (0.78-0.8) |
| Ethnicity | White | 1 (Ref) | 1 (Ref) | 1 (Ref) | 1 (Ref) |
|  | Mixed | 0.92 (0.83-1.02) | 0.91 (0.83-1.01) | 0.88 (0.8-0.98) | 0.92 (0.83-1.01) |
|  | Asian | 1.12 (1.09-1.15) | 1.12 (1.08-1.15) | 1.05 (1.02-1.08) | 1.13 (1.1-1.16) |
|  | Black | 0.89 (0.86-0.93) | 0.89 (0.85-0.93) | 0.85 (0.81-0.88) | 0.91 (0.88-0.95) |
|  | Other ethnic group | 0.92 (0.88-0.97) | 0.92 (0.87-0.96) | 0.9 (0.86-0.95) | 0.92 (0.88-0.97) |
|  | Not known | 1.02 (1-1.04) | 1.01 (0.99-1.04) | 1.01 (0.99-1.03) | 1.02 (0.99-1.04) |
| Admission method | Elective | 1 (Ref) | 1 (Ref) | 1 (Ref) | 1 (Ref) |
|  | Emergency | 2.01 (1.88-2.16) | 1.98 (1.84-2.12) | 1.96 (1.83-2.11) | 1.86 (1.73-1.99) |
|  | Maternity and Child | 0.43 (0.28-0.66) | 0.38 (0.25-0.58) | 0.43 (0.28-0.66) | 0.34 (0.23-0.52) |
|  | Transfers | 1.55 (1.43-1.68) | 1.54 (1.42-1.67) | 1.51 (1.4-1.64) | 1.52 (1.41-1.65) |
|  | Not known | 1.91 (0.93-3.91) | 1.91 (0.94-3.88) | 1.81 (0.88-3.72) | 1.83 (0.91-3.7) |
| Comorbidities | MI | 1 (0.98-1.03) | 1.13 (1.11-1.16) |  | 1.13 (1.11-1.15) |
|  | CCF | 1.28 (1.26-1.31) | 1.46 (1.44-1.49) |  | 1.47 (1.44-1.49) |
|  | PVD | 1.03 (1-1.06) | 1.16 (1.13-1.19) |  | 1.17 (1.14-1.2) |
|  | CVD | 0.94 (0.92-0.96) | 1.07 (1.05-1.09) |  | 1.08 (1.05-1.1) |
|  | DEMENTIA | 1.11 (1.09-1.13) | 1.29 (1.26-1.31) |  | 1.28 (1.26-1.31) |
|  | COPD | 1.05 (1.03-1.07) | 1.22 (1.2-1.24) |  | 1.21 (1.2-1.23) |
|  | CTD | 0.95 (0.92-0.99) | 1.09 (1.06-1.13) |  | 1.09 (1.05-1.12) |
|  | PUD | 0.92 (0.86-0.99) | 1.07 (1-1.15) |  | 1.08 (1.01-1.16) |
|  | HEMIPARA | 1.1 (1.05-1.15) | 1.37 (1.31-1.43) |  | 1.36 (1.3-1.42) |
|  | RENAL | 0.83 (0.82-0.85) | 1.07 (1.05-1.08) |  | 1.08 (1.07-1.1) |
|  | Diabetes | 0.87 (0.85-0.89) | 1.03 (1.01-1.04) |  | 1.03 (1.01-1.04) |
|  | Liver disease | 1.16 (1.12-1.2) | 1.43 (1.39-1.48) |  | 1.44 (1.4-1.49) |
|  | Cancer | 1.21 (1.18-1.25) | 1.68 (1.65-1.72) |  | 1.67 (1.64-1.71) |
| AKI |  | 2.24 (2.21-2.27) | 2.26 (2.23-2.29) | 2.19 (2.16-2.22) | 2.34 (2.31-2.37) |
| ITU |  | 6.34 (6.18-6.49) | 6.2 (6.05-6.35) | 6.28 (6.13-6.44) | 7.12 (6.96-7.29) |
| COVID variant | "Original" SARS CoV-2 variant | 1 (Ref) | 1 (Ref) | 1 (Ref) |  |
|  | "Alfa" SARS CoV-2 variant | 0.76 (0.75-0.78) | 0.77 (0.75-0.78) | 0.76 (0.75-0.77) |  |
| Acute dialysis |  | 2.99 (2.85-3.13) | 3.01 (2.87-3.15) | 2.89 (2.76-3.03) |  |
| CCI grades | No comorbidity | 1 (Ref) |  | 1 (Ref) |  |
|  | Mild comorbidities | 1.57 (1.53-1.61) |  | 1.6 (1.56-1.63) |  |
|  | Moderate comorbidities | 2.01 (1.94-2.09) |  | 1.97 (1.93-2.02) |  |
|  | Severe comorbidities | 2.52 (2.39-2.66) |  | 2.62 (2.55-2.69) |  |
| Deprivation deciles* | 10 | 1 (Ref) | 1 (Ref) | 1 (Ref) | 1 (Ref) |
|  | 9 | 1.04 (1.01-1.08) | 1.04 (1.01-1.08) | 1.04 (1-1.07) | 1.04 (1.01-1.08) |
|  | 8 | 1.05 (1.02-1.09) | 1.05 (1.02-1.09) | 1.05 (1.01-1.08) | 1.06 (1.02-1.09) |
|  | 7 | 1.05 (1.01-1.08) | 1.05 (1.01-1.08) | 1.04 (1-1.07) | 1.04 (1.01-1.08) |
|  | 6 | 1.06 (1.03-1.1) | 1.06 (1.03-1.09) | 1.05 (1.02-1.09) | 1.06 (1.02-1.09) |
|  | 5 | 1.08 (1.04-1.11) | 1.08 (1.04-1.11) | 1.06 (1.03-1.1) | 1.08 (1.04-1.11) |
|  | 4 | 1.08 (1.04-1.11) | 1.08 (1.04-1.11) | 1.06 (1.03-1.1) | 1.08 (1.04-1.11) |
|  | 3 | 1.1 (1.06-1.13) | 1.1 (1.06-1.13) | 1.09 (1.05-1.12) | 1.09 (1.06-1.13) |
|  | 2 | 1.06 (1.03-1.1) | 1.06 (1.03-1.1) | 1.05 (1.02-1.09) | 1.06 (1.03-1.1) |
|  | 1 | 1.1 (1.07-1.14) | 1.1 (1.07-1.14) | 1.08 (1.05-1.12) | 1.1 (1.07-1.14) |
| Region | London | 1 (Ref) | 1 (Ref) | 1 (Ref) | 1 (Ref) |
|  | North-East | 1.08 (1.05-1.12) | 1.09 (1.05-1.13) | 1.07 (1.04-1.11) | 1.08 (1.04-1.12) |
|  | North-West | 1.24 (1.21-1.27) | 1.25 (1.22-1.28) | 1.25 (1.22-1.28) | 1.24 (1.21-1.28) |
|  | Yorkshire & Humber | 1.16 (1.13-1.2) | 1.17 (1.13-1.2) | 1.17 (1.13-1.2) | 1.16 (1.12-1.19) |
|  | East Midlands | 1.15 (1.12-1.19) | 1.15 (1.12-1.19) | 1.16 (1.13-1.2) | 1.15 (1.11-1.18) |
|  | West Midlands | 1.12 (1.09-1.14) | 1.12 (1.09-1.15) | 1.12 (1.09-1.15) | 1.12 (1.09-1.15) |
|  | East of England | 1.24 (1.2-1.27) | 1.24 (1.21-1.28) | 1.24 (1.21-1.27) | 1.24 (1.2-1.27) |
|  | South-East | 1.15 (1.12-1.18) | 1.15 (1.12-1.18) | 1.15 (1.12-1.18) | 1.14 (1.11-1.17) |
|  | South-West | 1.06 (1.02-1.09) | 1.06 (1.02-1.1) | 1.04 (1.01-1.08) | 1.05 (1.01-1.09) |
| RECOVERY phases | Pre-RECOVERY "Original" SARS CoV-2 variant | 1 (Ref) | 1 (Ref) | 1 (Ref) | 1 (Ref) |
|  | Post-RECOVERY "Original" SARS CoV-2 variant | 0.73 (0.72-0.75) | 0.74 (0.72-0.75) | 0.73 (0.72-0.74) | 0.73 (0.71-0.74) |
|  | Post-RECOVERY "Alfa" SARS CoV-2 variant |  |  |  | 0.76 (0.74-0.77) |

^*^10 = least deprived

S6 Table: Sensitivity analysis for AKI predictors in patients hospitalised with COVID-19

|  |  | Odds ratio (95% CI) |
| --- | --- | --- |
| Age |  | 1.02 (1.02 -1.02) |
| Gender | Male | 1 (Ref) |
|  | Female | 0.78 (0.77 -0.79) |
|  | Other | 1.13 (0.85-1.5) |
| Ethnicity | White | 1 (Ref) |
|  | Mixed | 1.10 (1.02-1.17) |
|  | Asian | 0.92 (0.9-0.94) |
|  | Black | 1.47 (1.42-1.51) |
|  | Other ethnic group | 0.93 (0.9-0.97) |
|  | Not known | 1.03 (1.01-1.05) |
| Admission method | Elective | 1 (Ref) |
|  | Emergency | 1.98 (1.89-2.09) |
|  | Maternity and Child | 0.27 (0.22-0.33) |
|  | Transfers | 1.53 (1.44-1.62) |
|  | Not known | 0.85 (0.44-1.63) |
| Comorbidities | MI | 1.01 (0.99-1.03) |
|  | CCF | 1.36 (1.34-1.38) |
|  | PVD | 1.07 (1.04-1.09) |
|  | CVD | 0.97 (0.95-0.98) |
|  | DEMENTIA | 1.18 (1.17-1.2) |
|  | COPD | 0.92 (0.91-0.93) |
|  | CTD | 0.88 (0.85-0.9) |
|  | PUD | 1.31 (1.24-1.39) |
|  | HEMIPARA | 1.09 (1.05-1.13) |
|  | RENAL | 2.67 (2.63-2.7) |
|  | Diabetes | 1.24 (1.23-1.26) |
|  | Liver disease | 1.42 (1.38-1.45) |
|  | Cancer | 1.08 (1.06-1.1) |
|  |  | Odds ratio (95% CI) |
| Deprivation deciles^*^ | 10 | 1 (Ref) |
|  | 9 | 1.05 (1.02-1.08) |
|  | 8 | 1.03 (1-1.05) |
|  | 7 | 1.04 (1.02-1.07) |
|  | 6 | 1.08 (1.05-1.11) |
|  | 5 | 1.14 (1.11-1.17) |
|  | 4 | 1.1 (1.08-1.13) |
|  | 3 | 1.1 (1.07-1.12) |
|  | 2 | 1.07 (1.05-1.1) |
|  | 1 | 1.16 (1.13-1.2) |
| ITU |  | 3.36 (3.3-3.43) |
| RECOVERY phases | Pre-RECOVERY "Original" SARS CoV-2 variant | 1 (Ref) |
|  | Post-RECOVERY "Original" SARS CoV-2 variant | 0.86 (0.85-0.88) |
|  | Post-RECOVERY "Alfa" SARS CoV-2 variant | 0.86 (0.84-0.87) |
| Region | London | 1 (Ref) |
|  | North-East | 0.86 (0.84-0.89) |
|  | North-West | 0.76 (0.75-0.77) |
|  | Yorkshire & Humber | 0.79 (0.77-0.8) |
|  | East Midlands | 0.8 (0.78-0.82) |
|  | West Midlands | 0.78 (0.77-0.8) |
|  | East of England | 0.9 (0.88-0.92) |
|  | South-East | 0.87 (0.85-0.88) |
|  | South-West | 0.68 (0.66-0.69) |

^*^10 = least deprived

S1 Figure: Correlation between population density and population incidence of hospitalised COVID-19

S7 Table: STROBE Statement—checklist of items

|  | Item No. | Recommendation | | Page  No. | Relevant text from manuscript | |
| --- | --- | --- | --- | --- | --- | --- |
| **Title and abstract** | 1 | (*a*) Indicate the study’s design with a commonly used term in the title or the abstract | | 1 | Title | |
|  |  | (*b*) Provide in the abstract an informative and balanced summary of what was done and what was found | | 2 | Abstract | |
| Introduction | | | | |  |  |
| Background/rationale | 2 | Explain the scientific background and rationale for the investigation being reported | | 3-4 | Introduction | |
| Objectives | 3 | State specific objectives, including any prespecified hypotheses | | 4 | Introduction | |
| Methods | | | | |  |  |
| Study design | 4 | Present key elements of study design early in the paper | | 6 | Study design | |
| Setting | 5 | Describe the setting, locations, and relevant dates, including periods of recruitment, exposure, follow-up, and data collection | | 6-7 | Methods | |
| Participants | 6 | (*a*) *Cohort study*—Give the eligibility criteria, and the sources and methods of selection of participants. Describe methods of follow-up  *Case-control study*—Give the eligibility criteria, and the sources and methods of case ascertainment and control selection. Give the rationale for the choice of cases and controls  *Cross-sectional study*—Give the eligibility criteria, and the sources and methods of selection of participants | | 7 | Study design and data source | |
|  |  | (*b*) *Cohort study*—For matched studies, give matching criteria and number of exposed and unexposed  *Case-control study*—For matched studies, give matching criteria and the number of controls per case | |  |  | |
| Variables | 7 | Clearly define all outcomes, exposures, predictors, potential confounders, and effect modifiers. Give diagnostic criteria, if applicable | | 8 | Study design and procedures, definition | |
| Data sources/ measurement | 8* | For each variable of interest, give sources of data and details of methods of assessment (measurement). Describe comparability of assessment methods if there is more than one group | | *7* | Definition | |
| Bias | 9 | Describe any efforts to address potential sources of bias | | 8 | Statistical analysis | |
| Study size | 10 | Explain how the study size was arrived at | | 7 | Study design | |
| Quantitative variables | 11 | Explain how quantitative variables were handled in the analyses. If applicable, describe which groupings were chosen and why | 7 | | Definition |  |
| Statistical methods | 12 | (*a*) Describe all statistical methods, including those used to control for confounding | 8 | | Statistical analysis |  |
|  |  | (*b*) Describe any methods used to examine subgroups and interactions | 8 | | Statistical analysis |  |
|  |  | (*c*) Explain how missing data were addressed | 8 | | Statistical analysis |  |
|  |  | (*d*) *Cohort study*—If applicable, explain how loss to follow-up was addressed  *Case-control study*—If applicable, explain how matching of cases and controls was addressed  *Cross-sectional study*—If applicable, describe analytical methods taking account of sampling strategy |  | |  |  |
|  |  | (*e*) Describe any sensitivity analyses | 5,8 | | Statistical analysis, sults |  |
| Results | | | | | |  |
| Participants | 13* | (a) Report numbers of individuals at each stage of study—eg numbers potentially eligible, examined for eligibility, confirmed eligible, included in the study, completing follow-up, and analysed | 10 | | Results, figure 1 |  |
|  |  | (b) Give reasons for non-participation at each stage |  | | Figure 1 |  |
|  |  | (c) Consider use of a flow diagram |  | | Figure 1 |  |
| Descriptive data | 14* | (a) Give characteristics of study participants (eg demographic, clinical, social) and information on exposures and potential confounders | 10-17 | | Table 1a, 1b. Table 2, |  |
|  |  | (b) Indicate number of participants with missing data for each variable of interest | 8 | | Statistical analysis |  |
|  |  | (c) *Cohort study*—Summarise follow-up time (eg, average and total amount) |  | |  |  |
| Outcome data | 15* | *Cohort study*—Report numbers of outcome events or summary measures over time | 19-20 | | Table 3 |  |
|  |  | *Case-control study—*Report numbers in each exposure category, or summary measures of exposure |  | |  |  |
|  |  | *Cross-sectional study—*Report numbers of outcome events or summary measures |  | |  |  |
| Main results | 16 | (*a*) Give unadjusted estimates and, if applicable, confounder-adjusted estimates and their precision (eg, 95% confidence interval). Make clear which confounders were adjusted for and why they were included | 21-22 | | Figure 3a, 3b, 3c, 4a, 4b. |  |
|  |  | (*b*) Report category boundaries when continuous variables were categorized | 15,18 | | Table 1a, 1b and table 2 |  |
|  |  | (*c*) If relevant, consider translating estimates of relative risk into absolute risk for a meaningful time period |  | |  |  |
| Other analyses | 17 | Report other analyses done—eg analyses of subgroups and interactions, and sensitivity analyses | 12-22 | | Results |  |
| Discussion | | | | | |  |
| Key results | 18 | Summarise key results with reference to study objectives | 22-23 | | Discussion |  |
| Limitations | 19 | Discuss limitations of the study, taking into account sources of potential bias or imprecision. Discuss both direction and magnitude of any potential bias | 26 | | Discussion |  |
| Interpretation | 20 | Give a cautious overall interpretation of results considering objectives, limitations, multiplicity of analyses, results from similar studies, and other relevant evidence | 26-27 | | Conclusion |  |
| Generalisability | 21 | Discuss the generalisability (external validity) of the study results | 27 | | Conclusions |  |
| Other information |  | | | | |  |
| Funding | 22 | Give the source of funding and the role of the funders for the present study and, if applicable, for the original study on which the present article is based | 28 | | Role of funding source |  |

S1 Text - Protocol for Regional Epidemiology of COVID-19 and Acute Kidney Injury in England

| **FULL/ LONG TITLE OF THE STUDY**  Regional Epidemiology of COVID-19 and Acute Kidney Injury in England | |
| --- | --- |
| **SHORT TITLE**  **Re**gional **C**OVID **E**pi**d**emiology in **E**ngland (RECEDE) | |
| **Version and Date of Protocol:** | v1.2 9/SEPTEMBER/2020 |
| **Sponsor:** | University Hospitals of Derby & Burton NHS Foundation Trust |
| **Chief Investigator:** | Dr. Nitin V Kolhe |
| **Local Reference:** | UHDB/2020/075 |
| **IRAS Number:** | 287831 |
| **ISRCTN/ ClinicalTrials.gov number:** | NCT04579652 |
| **Funder(s):** | AWAITED |
| **This protocol has regard for the HRA guidance** | |

**Confidentiality Statement**

This document contains confidential information that must not be disclosed to anyone other than the Sponsor, the Investigator Team, host NHS Trust, regulatory authorities, and members of the Research Ethics Committee.

# SIGNATURE PAGE

The undersigned confirm that the following protocol has been agreed and accepted and that the Chief Investigator agrees to conduct the study in compliance with the approved protocol and will adhere to the principles outlined in the Declaration of Helsinki, the Sponsor’s SOPs, and other regulatory requirement.

I agree to ensure that the confidential information contained in this document will not be used for any other purpose other than the evaluation or conduct of the investigation without the prior written consent of the Sponsor.

I also confirm that I will make the findings of the study publically available through publication or other dissemination tools without any unnecessary delay and that an honest accurate and transparent account of the study will be given; and that any discrepancies from the study as planned in this protocol will be explained.

**Protocol v 1.2 9/SEPTEMBER/2020 authorisation signatures:**

| 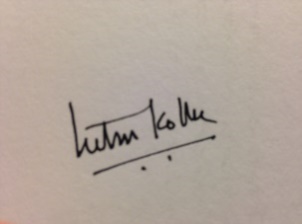**Chief Investigator:** | | | |
| --- | --- | --- | --- |
| Signature: | ………………………………… |  | Date:  9/SEPTEMBER/2020 |
| Name (please print): | Dr. Nitin Kolhe |  |  |
|  | | | |

# KEY STUDY CONTACTS

| Chief Investigator: | Nitin V Kolhe; 01332788266; [nitin.kolhe@nhs.net](mailto:nitin.kolhe@nhs.net) |
| --- | --- |
| Co-Investigator(s): | Maarten W Taal; 01332789344; [maarten.taal1@nhs.net](mailto:maarten.taal1@nhs.net) |
| Sponsor: | University Hospitals of Derby & Burton NHS Foundation Trust  Royal Derby Hospital  Uttoxeter Road  Derby, DE22 3NE  01332 724639  [UHDB.sponsor@nhs.net](mailto:UHDB.sponsor@nhs.net) |
| Funder(s): | None |

# STUDY SUMMARY

| **Study Title:** | Regional Epidemiology of COVID-19 and Acute Kidney Injury in England, United Kingdom |
| --- | --- |
| **Local Study Reference:** | UHDB/2020/050 |
| **Study Design:** | Retrospective cohort study |
| **Study Participants:** | All patients diagnosed of COVID-19 admitted to NHS hospital in England between 1^st^ March and 30^th^ June 2020 |
| **Planner Number of Sites:** | 1 |
| **Planned Sample Size:** | 5000 AKI patients |
| **Planned Start Date:** | 1^st^ October 2020 |
| **Planned Study End Date:** | 31^st^ August 2021 |
| **Research Question/ Aims:** | Epidemiology of AKI in patients hospitalised with COVID-19 in England, United Kingdom. |

# ROLES & RESPONSIBILITIES

**Sponsor**

The Sponsor, University Hospitals of Derby & Burton NHS Foundation Trust, take on overall responsibility for appropriate arrangements being in place to set up, run and report the research project. A Division of Responsibilities between the CI and Sponsor outlines the duties that have been delegated to the CI for this study.

**Study Management Committees**

No formal study management committees are required according to the Sponsor’s risk assessment for the study. The CI will provide status updates to the Sponsor according to their requirements.

Protocol Contributors

A number of protocol contributors have been involved in the development of this protocol, these include the Chief and the co-Investigator.

Protocol contributors are responsible for inputting into the design of the study, ensuring that it is designed transparently and efficiently.

Contents

[SIGNATURE PAGE 52](#_Toc39734959)

[KEY STUDY CONTACTS 53](#_Toc39734960)

[STUDY SUMMARY 54](#_Toc39734961)

[ROLES & RESPONSIBILITIES 55](#_Toc39734962)

[LIST OF ABBREVIATIONS 58](#_Toc39734963)

[STUDY FLOW CHART 59](#_Toc39734964)

[1. BACKGROUND 60](#_Toc39734965)

[2. RATIONALE 61](#_Toc39734966)

[3. OBJECTIVES AND OUTCOME MEASURES/ ENDPOINTS 61](#_Toc39734967)

[3.1. Objectives 61](#_Toc39734968)

[3.2. Outcome 61](#_Toc39734969)

[4. STUDY DESIGN 62](#_Toc39734970)

[5. STUDY SETTING 62](#_Toc39734971)

[6. ELIGIBILITY CRITERIA 62](#_Toc39734972)

[6.1. Inclusion Criteria 62](#_Toc39734973)

[6.2. Exclusion Criteria 63](#_Toc39734974)

[7. STUDY PROCEDURES 63](#_Toc39734975)

[7.1. Recruitment 63](#_Toc39734976)

[7.1.1. Patient Identification 63](#_Toc39734977)

[7.1.2. Screening 64](#_Toc39734978)

[7.2. Study Assessments 65](#_Toc39734979)

[7.3. End of Study 65](#_Toc39734980)

[8. DATA HANDLING 65](#_Toc39734981)

[8.1. System and Compliance 65](#_Toc39734982)

[8.2. Source Data 65](#_Toc39734983)

[8.3. Data Workflow 66](#_Toc39734984)

[8.4. Data Access and Security 66](#_Toc39734985)

[8.5. Archiving 66](#_Toc39734986)

[9. STATISTICS AND DATA ANALYSIS 66](#_Toc39734987)

[9.1. Sample Size Calculation 67](#_Toc39734988)

[9.2. Statistical Analysis 67](#_Toc39734992)

[9.2.1. Summary of Baseline Data and Flow of Patients 67](#_Toc39734993)

[9.2.2. Outcome Analysis 67](#_Toc39734994)

[9.2.3. Interim Analysis and Criteria for the Premature Termination of the Study 68](#_Toc39734995)

[9.2.4. Analysis Groups 68](#_Toc39734996)

[9.3. Procedure(s) to Account for Missing or Spurious Data 69](#_Toc39734997)

[10. MONITORING, AUDIT & INSPECTION 69](#_Toc39734998)

[11. ETHICAL AND REGULATORY CONSIDERATIONS 69](#_Toc39734999)

[11.1. Assessment and Management of Risk 69](#_Toc39735000)

[11.2. Peer review 69](#_Toc39735001)

[11.3. Public and Patient Involvement 69](#_Toc39735002)

[11.4. Research Ethics Committee (REC) & Regulatory Considerations 70](#_Toc39735003)

[11.5. Protocol Compliance / Non-compliance Reporting 70](#_Toc39735004)

[11.6. Notification of Serious Breaches to GCP and/or the Protocol 71](#_Toc39735005)

[11.7. Data Protection and Patient Confidentiality 71](#_Toc39735006)

[11.8. Financial and Other Competing Interests for the Chief Investigator, Principal Investigators at Each Site and Committee Members for the Overall Study Management 71](#_Toc39735007)

[11.9. Indemnity 71](#_Toc39735008)

[11.10. Amendments 72](#_Toc39735009)

[11.11. Access to Final Study Dataset 72](#_Toc39735010)

[12. DISSEMINATION POLICY 72](#_Toc39735011)

[12.1. Dissemination Policy 72](#_Toc39735012)

[12.2. Authorship Eligibility Guidelines and any Intended Use of Professional Writers 73](#_Toc39735013)

[13. APPENDICES 74](#_Toc39735014)

[13.1. Appendix 2 – Amendment History 74](#_Toc39735015)

# LIST OF ABBREVIATIONS

| AE | Adverse Event |
| --- | --- |
| CI | Chief Investigator |
| CRF | Case Report Form |
| DMEC | Data Monitoring and Ethics Committee |
| GCP | Good Clinical Practice |
| GMP | Good Manufacturing Practice |
| ICF | Informed Consent Form |
| ICH | International Conference on Harmonisation of technical requirements for registration of pharmaceuticals for human use. |
| ISF | Investigator Site File |
| ISRCTN | International Standard Randomised Controlled Trials |
| NHS R&D | National Health Service Research & Development |
| PI | Principal Investigator |
| PIC | Participant Identification Centre |
| PIS | Participant Information Sheet |
| QA | Quality Assurance |
| QC | Quality Control |
| RCT | Randomised Control Trial |
| REC | Research Ethics Committee |
| SAE | Serious Adverse Event |
| SDV | Source Data Verification |
| SOP | Standard Operating Procedure |
| TMG | Trial Management Group |
| TSC | Trial Steering Committee |
| TMF | Trial Master File |

STUDY FLOW CHART**
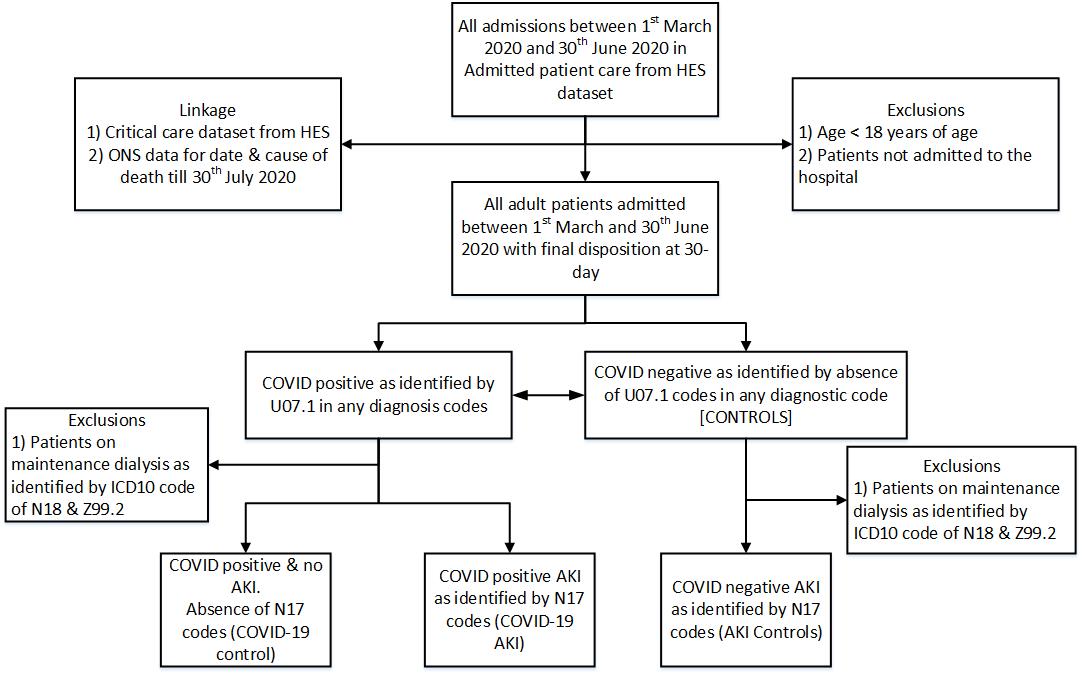
**

**STUDY PROTOCOL**

# BACKGROUND

The coronavirus disease 2019 (COVID-19) pandemic has caused significant morbidity and mortality and has affected some countries disproportionately. It has become evident that SARS-CoV-2 has shown variation in its virulence with some regions, within a country, more severely affected than others. In England, London and West Midlands were overwhelmed in the early period of the pandemic with increased in hospitalizations for pneumonia with multiorgan disease. As of 31^st^ July 2020, the number of confirmed cases for COVID-19 have exceeded 17 million world-wide and 300,000 in United Kingdom with 46,000 deaths in UK alone.[41] In UK, the pandemic reached its peak in mid-April with confirmed cases decreasing by end of May 2020.[42] One of the most serious complications of COVID-19 has been kidney involvement, in the form of acute kidney injury (AKI) but the incidence of this has ranged widely in reports from different centres, between 5% to 57%.[43-45] In the only report from UK, the incidence of AKI in COVID-19 was 26.2% which was much higher than a comparable cohort of people admitted with non-COVID acute illnesses (12.4%). However, no data have been published regarding variation in the incidence of COVID associated AKI within the UK. The medical community rapidly learnt lessons in the first wave of the pandemic, for example regarding the use of continuous positive pressure ventilation (CPAP) in case of respiratory distress and the use of regional anticoagulation in AKI needing continuous renal replacement therapy (CRRT).[46, 47] There was no effective treatment available at the start of the pandemic and this resulted in death rate rising sharply in England as well as many other countries. It has now become evident that there has been considerable variation in mortality in individual hospitals and there is urgent need to understand the reasons for this unwanted variation.[48] As the country tries to recover from the pandemic, it becomes more important to learn lessons with regards to its strategy of tackling the disease. This learning can then be applied to a possible second spike of COVID-19 to help mitigate the impact and improve outcomes.

To address this need for learning, we will combine a national database of hospital admissions and discharges with a critical care dataset and census data from office of national statistic (ONS) over a period of four months of the pandemic to investigate the regional incidence and mortality of patients with COVID-19 disease and acute kidney injury in England. We will also explore various determinants of mortality in patients admitted with COVID-19 and the impact of AKI on mortality.

# RATIONALE

The COVID-19 pandemic has exposed the unwanted variation in outcomes as evidence by Public Health England’s report on increased mortality in regions of the country. For example, UHDB, in East Midlands, has reported a high crude mortality as compared to other Trusts in the region.[48] There may also have been variation in the incidence of complications of COVID-19 in the form of AKI, which may have influenced mortality. Variation in outcomes may be because of various factors - differing population demographics, underlying health conditions in the population, deprivation, physician preference and knowledge and ethnic diversity. Unwanted variation is care that is not consistent with a patient’s preference or related to [their] underlying illness. It is important to understand the reason for unwanted variation in outcomes associated with COVID-19 to minimise patient harm and reduce morbidity and mortality.

# OBJECTIVES AND OUTCOME MEASURES/ ENDPOINTS

## Objectives

Primary objective

The primary objective is to describe the regional epidemiology of COVID-19 and AKI in hospitalised patients in England.

Secondary objectives:

To investigate the association between patient characteristics and patient outcomes in patients admitted with COVID-19 with or without AKI.

## Outcome

The primary outcomes are in-hospital all-cause mortality and incidence of AKI in patients with COVID-19.

Secondary outcomes will include, need for ventilatory support, admission to ICU, length of stay.

# STUDY DESIGN

This is a retrospective, cohort study of hospitalised COVID-19 patients identified from national data collected during routine care in hospital settings. The study will have two work packages. Work package 1 (WP1) will investigate the regional variation of in-hospital mortality in COVID-19 and its associated predictors, including AKI. Work package 2 (WP2) will investigate the regional variation in the incidence of COVID associated AKI and its associated predictors. WP2 will further investigate differences between COVID associated AKI and AKI due to other acute illnesses.

# STUDY SETTING

This is a retrospective, cohort study of hospitalised COVID-19 patients in England between 1^st^ March 2020 and 30^th^ June 2020 from hospital episode statistic (HES) dataset of NHS Digital.

# ELIGIBILITY CRITERIA

## Inclusion Criteria

This study will have two work packages.

**Work Package 1 (WP1): Regional epidemiology of COVID-19 in England**

All patients who are admitted to hospital with COVID-19 infection between 1^st^ March 2020 and 30^th^ June 2020 and who meet following criteria will be included

1. Diagnostic code for COVID-19 (U07.1) in any of the 20 diagnoses codes
2. Adult patients greater or equal to 18 years of age.

**Work Package 2 (WP2): Epidemiology of COVID-19 associated AKI in England**

All patients who are admitted to hospital with acute kidney injury (AKI) between 1^st^ March 2020 and 30^th^ June 2020 and who meet following criteria will be included

1. Diagnostic code for AKI (N17) in any of the 20 diagnoses codes
2. Adult patients greater or equal to 18 years of age.

## Exclusion Criteria

**WP1:**

The following patients will be excluded from the study

1. Patients not admitted to the hospital
2. Paediatric patients < 18 years of age

**WP2:**

The following patients will be excluded from the study

1. Patients not admitted to the hospital
2. Paediatric patients < 18 years of age
3. Patients on chronic maintenance haemodialysis or peritoneal dialysis

# STUDY PROCEDURES

## Recruitment

### Patient Identification

In WP1 we will identify all cases of COVID-19 by using validated International Classification of Diseases, Tenth Revision, Clinical Modification (ICD-10-CM) code of U07.1 in any of the diagnoses codes, during the study period.[49]

In WP2, patients with following ICD-10 codes for acute kidney injury, during the study period, will be included: N17.0 for acute renal failure with tubular necrosis, N17.1 for acute renal failure with acute cortical necrosis, N17.2 for acute renal failure with medullary necrosis, N17.8 for other acute renal failure and N17.9 for acute renal failure, unspecified. Acute renal failure has been replaced by new terminology, acute kidney injury, but due to lack of ICD10 codes for AKI, we will use the ICD10 codes for acute renal failure and henceforth, will be referred to as AKI in this protocol. Within the cohort of AKI patients, COVID-19 will be identified by ICD-10 code of U07.1 in any of the diagnostic codes and this will be the COVID-19 AKI cohort. The AKI cohort without COVID-19 will serve as AKI controls. The control group in the same period removes selection bias as all causes of AKI would have received the same level of diagnostic and therapeutic care.

### Screening

We will extract data of all admitted patients in England between 1^st^ March 2020 and 30^th^ June 2020 from Hospital Episode Statistics (HES), a data warehouse containing details of all admissions, outpatient appointments and A&E attendances at NHS hospitals in England. HES collects a detailed record for each 'episode' of admitted patient care delivered in England, either by NHS hospitals, primary care trusts, mental health trusts or delivered in the independent sector but commissioned by the NHS. These data are collected during a patient's time in hospital and stored as a large collection of separate records, one for each period of care, in a secure data warehouse. In addition to the codes for COVID-19 and AKI (described above in section 7.1.1.), we will extract 20 secondary diagnosis codes to assess prevalent comorbidities. Further, we will extract demographic data and up to 25 codes for procedures which patients underwent during their hospital stay. AKI patients requiring dialysis (AKI-D) will be identified by the additional procedure code of X40.2 for peritoneal dialysis, X40.3 for haemodialysis , X40.4 for hemofiltration, X405 for automated peritoneal dialysis or X40.6 for continuous ambulatory peritoneal dialysis in any of the 25 procedures. To exclude patients with End Stage kidney disease starting dialysis, we will exclude patients who had following codes: N18 – End Stage Renal Disease, Z99.2 – dependence on renal dialysis or L74.2 - arteriovenous fistula during the inpatient admission. This algorithm has been shown to be sensitive and specific, with a high positive and negative predictive value (all >90%)[50].

For both work packages, we will obtain data on patient demographics, hospital characteristics, in-hospital mortality, disposition, length of stay (LOS), and up to 20 diagnosis and 25 procedure codes that are based on the ICD-10-CM and OPCS-4, region, place of care and deprivation decile from the HES database. Data for critical care admission, discharge and organ support will be obtained from the linked critical care dataset. Patient’s status at the time of data extraction will be obtained from the Office of National Statistics (ONS). Patients will not be contacted. This study will not obtain informed consent from the patients as we will be working with anonymous patient data. No screening requirements (laboratory or diagnostic testing) are necessary to meet the inclusion or exclusion criteria. Unique subject Identification numbers are already allocated to the patients and the HES database does not include any patient’s name or hospital number.

## Study Assessments

As this is a retrospective study from the HES dataset, no regular study assessments will be performed.

## End of Study

The end date for the study will be 31^st^ August 2021

# DATA HANDLING

## System and Compliance

Anonymised data will be extracted in excel or csv format and will be stored on encrypted and password protected computer. The data will be checked for errors and duplicates and once accuracy is confirmed, a final version will be used for analysis.

## Source Data

The data source will be the HES data warehouse of NHS Digital. Data quality reports and checks are completed at various stages in the cleaning and processing cycle, and any duplication is removed.

## Data Workflow

The data will be extracted from HES data warehouse and will be linked to critical care dataset and ONS data. The data will be hosted on a Trust computer with password protected encryption. The data quality will be checked to ensure validity and the chief investigator will be responsible for the data security.

## Data Access and Security

Anonymised data will be stored on an encrypted computer of the chief investigator which is password protected. Direct access will be granted to authorised representatives from the Sponsor, host institution and the regulatory authorities to permit study-related monitoring, audits and inspections. Data will not be transferred outside of the UK.

## Archiving

At the end of the study, following completion of the end of study report, UHDB will securely archive all centrally held study related documentation for a minimum of 5 years. At the end of the defined archive period arrangements for confidential destruction will be made. The data and all essential documents relating to the study will be retained securely for a minimum of 5 years after the end of study, and in accordance with national legislation. All archived documents will continue to be available for inspection by appropriate authorities upon request.

# STATISTICS AND DATA ANALYSIS

WP1:

1. The primary analysis will be to describe the regional mortality in patients with COVID-19.
2. The secondary analysis will evaluate the predictors of mortality in COVID-19 patients.

WP2:

1. The primary analysis will be to describe the regional incidence of AKI in patients with COVID-19.
2. Secondary analyses will:
   1. Evaluate the predictors of AKI in COVID-19
   2. Evaluate the predictors of mortality in AKI patients

Sample Size Calculation

This is a retrospective, non-CTIMP cohort study and randomization or related processes are not applicable. A recent single-center study performed at UHDB, identified 300 cases of AKI between 5^th^ March and 12^th^ May. This provided a sufficient sample size to evaluate all of the outcomes proposed in this study. There are 209 acute NHS Trusts in England and we expect minimum 5000 cases of AKI in the four month study period.

## Statistical Analysis

### Summary of Baseline Data and Flow of Patients

We will present demographic and clinical characteristics of patients with COVID-19 according to English regions as described by Office of National Statistics. Government offices for the regions (GOR) were established across England in 1994. In 1996. the GORs became the primary classification for the presentation of regional statistics and are divided as North East, North West, South East, South West, East Midlands, West Midlands, East of England, Yorkshire and Humber and London. Descriptive statistics will be presented to summarize the distribution of baseline variables across the groups. The continuous baseline variables will be reported with means & 95% confidence intervals (95% CI), if shown to be normally distributed will be reported with medians & Interquartile Ranges (IQR). The categorical variables will be reported with frequencies & percentages.

### Outcome Analysis

WP1. Univariate analysis will be performed to ascertain any significant association of baseline variables with mortality in patients with COVID-19 disease. Any variables with significant association in univariate analysis or biological plausibility will be entered into a multivariable logistic regression analysis to ascertain the effects of age, gender, ethnicity, comorbidities, use of critical care, organ supported, region and acute kidney injury on the likelihood of in-hospital death.

WP2. Univariate analysis will be performed to ascertain any significant association of baseline variables with AKI in patients with COVID-19 disease. Any variables with significant association in univariate analysis or biological plausibility will be entered into a multivariable logistic regression analysis to ascertain the effects of age, gender, ethnicity, comorbidities, use of critical care, organ supported and region on the likelihood of AKI. A further analysis will include all patients (with or without COVID) who developed AKI during the study period. Univariate analysis will be performed to ascertain any significant association of baseline variables with mortality in all patients with AKI. Any variables with significant association in univariate analysis or biological plausibility will be entered into a multivariable logistic regression analysis to ascertain the effects of age, gender, ethnicity, comorbidities, use of critical care, organ supported, region and COVID-19 on the likelihood of in-hospital death.

All tests will be two-tailed, and p<0.05 will be considered significant. Analysis will be performed on IBM SPSS Statistics for Mac, Version 24.0

### Interim Analysis and Criteria for the Premature Termination of the Study

This is not applicable to a retrospective cohort study

### Analysis Groups

We will analyse the following groups:

1. Patients admitted to hospital with COVID-19.
2. Patients admitted to hospital with COVID-19 who also developed AKI during their hospital stay, referred as “COVID-19 AKI”.
3. Patients admitted to hospital with COVID-19 who did not develop AKI during their hospital stay, referred as “COVID-19 controls”.
4. Patients admitted to hospital with an acute illness who also developed AKI during their hospital stay but who did not have COVID-19, referred as “AKI controls”.

## Procedure(s) to Account for Missing or Spurious Data

The data will be checked for errors and depending on the amount of missing data, imputation may be performed

# MONITORING, AUDIT & INSPECTION

The investigator will permit study-related monitoring, audits and inspections by the Sponsor as required. This study will not be routinely monitored by the Sponsor, in accordance to the low risk nature of the study, but may be subject to audit for the QA team within the R&D department.

.

# ETHICAL AND REGULATORY CONSIDERATIONS

## Assessment and Management of Risk

As part of the Sponsor’s review process for all research projects, the study has been risk assessed according to their SOP. This study has been assessed as low risk.

## Peer review

The study protocol was peer reviewed by Prof. Maarten Taal, co-investigator of the study.

## Public and Patient Involvement

Patients have been involved in the design of protocol and will be involved in result and dissemination of the findings

## Research Ethics Committee (REC) & Regulatory Considerations

The study will be conducted in compliance with the approved protocol and the Declaration of Helsinki. Research limited to secondary use of information previously collected in the course of normal care (without an intention to use it for research at the time of collection) is generally excluded from REC review, provided that the patients or service users are not identifiable to the research team in carrying out the research. The investigator will not begin any participant activities until approval from the HRA has been obtained and documented. All documentation and correspondence must be retained in the trial master file/investigator site file. Substantial amendments that require HRA and REC (where applicable) review will not be implemented until the HRA and REC grants a favourable opinion (with the exception of those necessary to reduce immediate risk to participants).

It is the responsibility of the CI to ensure that an annual progress report (APR) is submitted to the REC (where applicable) within 30 days of the anniversary date on which the favourable opinion was given, annually until the study is declared ended. The CI is also responsible for notifying the REC of the end of study (see Section 6.9) within 90 days. Within one year of the end of study, the CI will submit a final report with the results, including any publications/abstracts to the REC.

Before any site can enroll a patient into the study confirmation of capacity must be sought from the site’s research and development (R&D) department. In addition for any amendment that will potentially affect the site’s permission, the research team must confirm with the site’s R&D department that permission is ongoing (Section 11.10).

## Protocol Compliance / Non-compliance Reporting

The investigator is responsible for ensuring that the study is conducted in accordance with the procedures described in this protocol. Prospective, planned deviations and/or waivers to the protocol are not acceptable, however accidental protocol deviations (non-compliances) may happen and as such these must be recorded. Non-compliances should be recorded in the CRF and/or a non-compliance log kept in the ISF. All non-compliances should be reviewed and assessed by the PI (or appropriately delegated individual) to determine if they meet the criteria of a “serious breach” (Section 12.6). Non-compliances which are found to frequently recur are not acceptable, will require immediate action, and could potentially be classified as a serious breach.

## Notification of Serious Breaches to GCP and/or the Protocol

A “serious breach” is a departure from the protocol, Sponsor procedures (i.e. SOPs), or regulatory requirements which is likely to effect to a significant degree –

- 1. The safety or physical or mental integrity of the subjects of the study; or
  2. The scientific value of the study.

If the PI (or delegate) is unsure if a non-compliance meets these criteria, they should consult the Sponsor for further guidance.

If a serious breach is identified the investigator should notify the Sponsor immediately (i.e. within 1 working day) using the ‘Non-CTIMP Notification of a Serious Breach’ form. The report will be reviewed by the Sponsor and CI, and where appropriate, the Sponsor will notify the REC within 7 calendar days of being made aware of the breach.

## Data Protection and Patient Confidentiality

The study will be conducted in accordance with the Data Protection Act 2018. The investigator will ensure that participant’s anonymity is maintained throughout the study and following completion of the study. The anonymised dataset obtained from NHS digital will not contain any patient identifiable data.

All documents will be stored securely with access restricted to study staff and authorised personnel.

Nitin V Kolhe will act as the custodian of the data generated in the study.

## Financial and Other Competing Interests for the Chief Investigator, Principal Investigators at Each Site and Committee Members for the Overall Study Management

The researchers declare no conflict of financial or other competing interest

## Indemnity

As UHDB is acting as the research Sponsor for this study, NHS indemnity applies. NHS indemnity provides cover for legal liabilities where the NHS has a duty of care. Non-negligent harm is not covered by the NHS indemnity scheme. UHDB, therefore, cannot agree in advance to pay compensation in these circumstances. In exceptional circumstances an ex-gratia payment may be offered.

## Amendments

If changes to the study are required these must be discussed with the Sponsor, who is responsible for deciding if an amendment is required and if it should be deemed substantial or non-substantial. Substantial amendments will be submitted to the relevant regulatory bodies (REC, HRA) for review and approval. The amendments will only be implemented after approval and a favourable opinion has been obtained. Non-substantial amendments will be submitted to the HRA for their approval/ acknowledgment. Amendments will not be implemented until all relevant approvals are in place.

## Access to Final Study Dataset

Dr. Nitin Kolhe will have the access to final dataset which will be received in anonymised form from NHS Digital.

# DISSEMINATION POLICY

## Dissemination Policy

The data is owned by UHDB NHS Trust and on completion of the study, the data will be analysed and tabulated and a Final Study Report prepared.

- The full study report can be accessed from the chief investigator.
- Participating investigators will have rights to publish any of the study data after discussion with the chief investigator.
- UHDB NHS Trust who sponsored the study will be acknowledged within the publications.
- The study protocol, full study report, pseudo-anonymised participant level dataset, and statistical code for generating the results will be made publicly available on clinicaltrials.gov after ethics approval.
- The study will be reported using the STROBE checklist for observational studies
- Limitations of the study include –
  - Retrospective nature of the study
  - No urinary chemistry will be available
  - Sensitivity and specificity of ICD codes for COVID-19 has not been examined
  - Sensitivity of ICD-10 codes for AKI will be lower than that recognised by NHS England’s AKI algorithm
  - The current proposed analysis of risk factors for AKI doesn’t analyse risks and interpretations will be limited to descriptive associations.
  - Other potentials for bias from:
    - Omitting patients without 30-day disposition
    - Missing data
    - Mortality estimated at 30 days
  - Unmeasured confounders (eg underlying health conditions, deprivation, physician preference, resource availability etc) will are likely to be an important limitation

## Authorship Eligibility Guidelines and any Intended Use of Professional Writers

Authorship will be provided as per the International Committee of Medical Journal Editors definition of authorship criteria for manuscripts submitted for publication.

# APPENDICES

## Appendix 2 – Amendment History

| **Amendment No.** | **Protocol version no.** | **Date issued** | **Author(s) of changes** | **Details of changes made** |
| --- | --- | --- | --- | --- |
|  |  |  |  |  |

Detail all protocol amendments. Protocol amendments must be submitted to the Sponsor for approval prior to submission to the REC.

**References**

1. Cereda D, Manica M, Tirani M, Rovida F, Demicheli V, Ajelli M, Poletti P, Trentini F, Guzzetta G, Marziano V *et al*: **The early phase of the COVID-19 epidemic in Lombardy, Italy**. *Epidemics* 2021, **37**:100528.

2. Strully K, Yang TC, Liu H: **Regional variation in COVID-19 disparities: connections with immigrant and Latinx communities in U.S. counties**. *Ann Epidemiol* 2021, **53**:56-62 e52.

3. Rideout A, Murray C, Isles C: **Regional variation in COVID-19 positive hospitalisation across Scotland during the first wave of the pandemic and its relation to population density: A cross-sectional observation study**. *PLoS One* 2021, **16**(7):e0253636.

4. Gray WK, Navaratnam AV, Day J, Wendon J, Briggs TWR: **Changes in COVID-19 in-hospital mortality in hospitalised adults in England over the first seven months of the pandemic: An observational study using administrative data**. *Lancet Reg Health Eur* 2021, **5**:100104.

5. Kulu H, Dorey P: **Infection rates from Covid-19 in Great Britain by geographical units: A model-based estimation from mortality data**. *Health Place* 2021, **67**:102460.

6. Morrissey K, Spooner F, Salter J, Shaddick G: **Area level deprivation and monthly COVID-19 cases: The impact of government policy in England**. *Soc Sci Med* 2021, **289**:114413.

7. Daras K, Alexiou A, Rose TC, Buchan I, Taylor-Robinson D, Barr B: **How does vulnerability to COVID-19 vary between communities in England? Developing a Small Area Vulnerability Index (SAVI)**. *J Epidemiol Community Health* 2021, **75**(8):729-734.

8. Kontopantelis E, Mamas MA, Deanfield J, Asaria M, Doran T: **Excess mortality in England and Wales during the first wave of the COVID-19 pandemic**. *J Epidemiol Community Health* 2021, **75**(3):213-223.

9. McNamara S, Holmes J, Stevely AK, Tsuchiya A: **How averse are the UK general public to inequalities in health between socioeconomic groups? A systematic review**. *Eur J Health Econ* 2020, **21**(2):275-285.

10. Irizar P, Pan D, Kapadia D, Becares L, Sze S, Taylor H, Amele S, Kibuchi E, Divall P, Gray LJ *et al*: **Ethnic inequalities in COVID-19 infection, hospitalisation, intensive care admission, and death: a global systematic review and meta-analysis of over 200 million study participants**. *EClinicalMedicine* 2023, **57**:101877.

11. Mahajan UV, Larkins-Pettigrew M: **Racial demographics and COVID-19 confirmed cases and deaths: a correlational analysis of 2886 US counties**. *J Public Health (Oxf)* 2020, **42**(3):445-447.

12. Varsavsky T, Graham MS, Canas LS, Ganesh S, Capdevila Pujol J, Sudre CH, Murray B, Modat M, Jorge Cardoso M, Astley CM *et al*: **Detecting COVID-19 infection hotspots in England using large-scale self-reported data from a mobile application: a prospective, observational study**. *Lancet Public Health* 2021, **6**(1):e21-e29.

13. Munford L, Khavandi S, Bambra C: **COVID-19 and deprivation amplification: An ecological study of geographical inequalities in mortality in England**. *Health Place* 2022, **78**:102933.

14. Barnard S, Fryers P, Fitzpatrick J, Fox S, Waller Z, Baker A, Burton P, Newton J, Doyle Y, Goldblatt P: **Inequalities in excess premature mortality in England during the COVID-19 pandemic: a cross-sectional analysis of cumulative excess mortality by area deprivation and ethnicity**. *BMJ Open* 2021, **11**(12):e052646.

15. Welsh C, Albani V, Matthews F, Bambra C: **Inequalities in the evolution of the COVID-19 pandemic: an ecological study of inequalities in mortality in the first wave and the effects of the first national lockdown in England**. *BMJ Open* 2022, **12**(8):e058658.

16. Gray WK, Navaratnam AV, Day J, Babu P, Mackinnon S, Adelaja I, Bartlett-Pestell S, Moulton C, Mann C, Batchelor A *et al*: **Variability in COVID-19 in-hospital mortality rates between national health service trusts and regions in England: A national observational study for the Getting It Right First Time Programme**. *EClinicalMedicine* 2021, **35**:100859.

17. Gray WK, Navaratnam AV, Day J, Wendon J, Briggs TWR: **COVID-19 hospital activity and in-hospital mortality during the first and second waves of the pandemic in England: an observational study**. *Thorax* 2022, **77**(11):1113-1120.

18. Huang YQ, Gou R, Diao YS, Yin QH, Fan WX, Liang YP, Chen Y, Wu M, Zang L, Li L *et al*: **Charlson comorbidity index helps predict the risk of mortality for patients with type 2 diabetic nephropathy**. *J Zhejiang Univ Sci B* 2014, **15**(1):58-66.

19. **Regional and sub-regional estimates of coronavirus (COVID-19) positivity over time, UK: 12 January 2023**

20. Horby P, Lim WS, Emberson JR, Mafham M, Bell JL, Linsell L, Staplin N, Brightling C, Ustianowski A, Elmahi E *et al*: **Dexamethasone in Hospitalized Patients with Covid-19**. *N Engl J Med* 2021, **384**(8):693-704.

21. Sun Z, Zhang H, Yang Y, Wan H, Wang Y: **Impacts of geographic factors and population density on the COVID-19 spreading under the lockdown policies of China**. *Sci Total Environ* 2020, **746**:141347.

22. Tammes P: **Social distancing, population density, and spread of COVID-19 in England: a longitudinal study**. *BJGP Open* 2020, **4**(3).

23. Buchan IE, Kontopantelis E, Sperrin M, Chandola T, Doran T: **North-South disparities in English mortality1965-2015: longitudinal population study**. *J Epidemiol Community Health* 2017, **71**(9):928-936.

24. Jha AK, Li Z, Orav EJ, Epstein AM: **Care in U.S. hospitals--the Hospital Quality Alliance program**. *N Engl J Med* 2005, **353**(3):265-274.

25. Asch DA, Sheils NE, Islam MN, Chen Y, Werner RM, Buresh J, Doshi JA: **Variation in US Hospital Mortality Rates for Patients Admitted With COVID-19 During the First 6 Months of the Pandemic**. *JAMA Intern Med* 2021, **181**(4):471-478.

26. [**https://www.nimblefins.co.uk/hospital-beds-uk-region-covid-19**](https://www.nimblefins.co.uk/hospital-beds-uk-region-covid-19)

27. Batchelor G: [**https://www.hsj.co.uk/quality-and-performance/revealed-huge-regional-variation-in-nhs-ability-to-meet-coronavirus-demand/7027153.article**](https://www.hsj.co.uk/quality-and-performance/revealed-huge-regional-variation-in-nhs-ability-to-meet-coronavirus-demand/7027153.article). *Health Service Journal* 2020.

28. Williamson EJ, Walker AJ, Bhaskaran K, Bacon S, Bates C, Morton CE, Curtis HJ, Mehrkar A, Evans D, Inglesby P *et al*: **Factors associated with COVID-19-related death using OpenSAFELY**. *Nature* 2020, **584**(7821):430-436.

29. Aldridge RW, Lewer D, Katikireddi SV, Mathur R, Pathak N, Burns R, Fragaszy EB, Johnson AM, Devakumar D, Abubakar I *et al*: **Black, Asian and Minority Ethnic groups in England are at increased risk of death from COVID-19: indirect standardisation of NHS mortality data**. *Wellcome Open Res* 2020, **5**:88.

30. Sze S, Pan D, Nevill CR, Gray LJ, Martin CA, Nazareth J, Minhas JS, Divall P, Khunti K, Abrams KR *et al*: **Ethnicity and clinical outcomes in COVID-19: A systematic review and meta-analysis**. *EClinicalMedicine* 2020, **29**:100630.

31. Mathur R, Rentsch CT, Morton CE, Hulme WJ, Schultze A, MacKenna B, Eggo RM, Bhaskaran K, Wong AYS, Williamson EJ *et al*: **Ethnic differences in SARS-CoV-2 infection and COVID-19-related hospitalisation, intensive care unit admission, and death in 17 million adults in England: an observational cohort study using the OpenSAFELY platform**. *Lancet* 2021, **397**(10286):1711-1724.

32. Kolhe NV, Fluck RJ, Muirhead AW, Taal MW: **Regional Variation in Acute Kidney Injury Requiring Dialysis in the English National Health Service from 2000 to 2015 - A National Epidemiological Study**. *PLoS One* 2016, **11**(10):e0162856.

33. Hassan MO, Balogun RA: **The Effects of Race on Acute Kidney Injury**. *J Clin Med* 2022, **11**(19).

34. Neugarten J, Golestaneh L, Kolhe NV: **Sex differences in acute kidney injury requiring dialysis**. *BMC Nephrol* 2018, **19**(1):131.

35. Kolhe NV, Muirhead AW, Wilkes SR, Fluck RJ, Taal MW: **The epidemiology of hospitalised acute kidney injury not requiring dialysis in England from 1998 to 2013: retrospective analysis of hospital episode statistics**. *Int J Clin Pract* 2016, **70**(4):330-339.

36. Kolhe NV, Muirhead AW, Wilkes SR, Fluck RJ, Taal MW: **National trends in acute kidney injury requiring dialysis in England between 1998 and 2013**. *Kidney Int* 2015, **88**(5):1161-1169.

37. [**https://www.england.nhs.uk/statistics/statistical-work-areas/covid-19-vaccinations/**](https://www.england.nhs.uk/statistics/statistical-work-areas/covid-19-vaccinations/)

38. Ho KS, Narasimhan B, Difabrizio L, Rogers L, Bose S, Li L, Chen R, Sheehan J, El-Halabi MA, Sarosky K *et al*: **Impact of corticosteroids in hospitalised COVID-19 patients**. *BMJ Open Respir Res* 2021, **8**(1).

39. van Paassen J, Vos JS, Hoekstra EM, Neumann KMI, Boot PC, Arbous SM: **Corticosteroid use in COVID-19 patients: a systematic review and meta-analysis on clinical outcomes**. *Crit Care* 2020, **24**(1):696.

40. Bagshaw SM, Goldstein SL, Ronco C, Kellum JA, Group AC: **Acute kidney injury in the era of big data: the 15(th) Consensus Conference of the Acute Dialysis Quality Initiative (ADQI)**. *Can J Kidney Health Dis* 2016, **3**:5.

41. [**https://www.ecdc.europa.eu/en/geographical-distribution-2019-ncov-cases**](https://www.ecdc.europa.eu/en/geographical-distribution-2019-ncov-cases)

42. [**https://coronavirus-staging.data.gov.uk/cases**](https://coronavirus-staging.data.gov.uk/cases)

43. Cheng Y, Luo R, Wang K, Zhang M, Wang Z, Dong L, Li J, Yao Y, Ge S, Xu G: **Kidney disease is associated with in-hospital death of patients with COVID-19**. *Kidney Int* 2020, **97**(5):829-838.

44. Hirsch JS, Ng JH, Ross DW, Sharma P, Shah HH, Barnett RL, Hazzan AD, Fishbane S, Jhaveri KD, Northwell C-RC *et al*: **Acute Kidney Injury in Patients Hospitalized with Covid-19**. *Kidney Int* 2020.

45. Pei G, Zhang Z, Peng J, Liu L, Zhang C, Yu C, Ma Z, Huang Y, Liu W, Yao Y *et al*: **Renal Involvement and Early Prognosis in Patients with COVID-19 Pneumonia**. *J Am Soc Nephrol* 2020.

46. [**https://www.nice.org.uk/guidance/ng175**](https://www.nice.org.uk/guidance/ng175)

47. [**https://www.nice.org.uk/guidance/ta139/chapter/1-guidance**](https://www.nice.org.uk/guidance/ta139/chapter/1-guidance)

48. [**https://www.england.nhs.uk/statistics/statistical-work-areas/covid-19-hospital-activity/**](https://www.england.nhs.uk/statistics/statistical-work-areas/covid-19-hospital-activity/)

49. [**https://www.who.int/classifications/icd/covid19/en/**](https://www.who.int/classifications/icd/covid19/en/)

50. Waikar SS, Wald R, Chertow GM, Curhan GC, Winkelmayer WC, Liangos O, Sosa MA, Jaber BL: **Validity of International Classification of Diseases, Ninth Revision, Clinical Modification Codes for Acute Renal Failure**. *J Am Soc Nephrol* 2006, **17**(6):1688-1694.
